# Ruthenium-enhanced curcumin derivatives target tumor growth and cancer-related inflammation in head and neck cancer models

**DOI:** 10.3389/fonc.2025.1708944

**Published:** 2025-12-17

**Authors:** Kateřina Veselá, Ameneh Tatar, Zdeněk Kejík, Nikita Abramenko, Robert Kaplánek, Petr Babula, Kateřina Kučnirová, Jan Hajduch, Pavel Martásek, Milan Jakubek

**Affiliations:** 1 BIOCEV, Biotechnology and Biomedicine Center of the Academy of Sciences and Charles University in Vestec, Vestec, Czechia; 2 Department of Paediatrics and Inherited Metabolic Disorders, First Faculty of Medicine, Charles University and General University Hospital in Prague, Prague, Czechia; 3 Department of Pathological Physiology, Faculty of Medicine, Masaryk University, Brno, Czechia; 4 Department of Physiology, Faculty of Medicine, Masaryk University, Brno, Czechia

**Keywords:** Ru-complex, curcumin, anti-inflammatory, anticancer, head and neck carcinoma

## Abstract

**Introduction:**

Head and neck cancers (HNC) remain a significant clinical challenge, particularly due to their association with chronic inflammation triggered by tobacco carcinogens and human papillomavirus (HPV) infection. Persistent activation of proinflammatory and proangiogenic pathways, including nuclear factor kappa B (NF-kB), interleukin 6 (IL-6), and interleukin 8 (IL-8), plays a crucial role in tumor progression.

**Methods:**

In this study, we synthetized ruthenium-enhanced curcumin derivatives (complexes 3 and 4) and study their anti-inflammatory and anticancer properties by using HNC cell lines.

**Results:**

Complex 3 demonstrated potent cytotoxic and antiproliferative effects across both HPV-negative and HPV- positive HNC cell lines, while complex 4 showed selectivity toward oral squamous cell carcinoma (OSCC). Both complexes exhibited cytostatic and migrastatic activities. Importantly, treatment with these complexes significantly suppressed NF-kB activity and reduced IL-6 and IL-8 levels more effectively than native curcumin.

**Discussion:**

These findings highlight their potential not only as stand-alone therapeutic agents but also as adjuvants in combination therapies for HNC.

## Graphical Abstract

**Figure:**
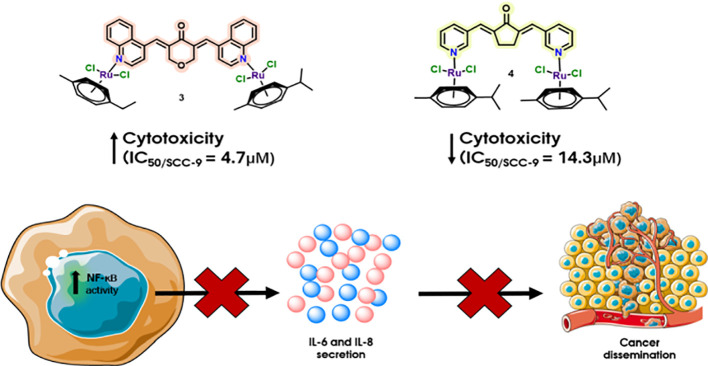

## 1 Introduction

Head and neck squamous cell carcinoma (HNSCC) includes a broad range of tumors that originate from the mucosa of the oral cavity, nasal cavity, larynx, salivary glands, and other associated sites (1–3). Among the primary etiological factors contributing to the development of head and neck squamous cell carcinoma are lifestyle-related exposures, particularly tobacco smoking and alcohol consumption (4, 5). Cigarette smoke has been shown to activate the transcription factor nuclear factor kappa B (NF-κB), which plays a central role in regulating inflammatory signaling pathways. NF-κB activation is associated with enhanced metastatic potential, tumor cell survival, and decreased sensitivity to chemoradiotherapy (6–8). In addition, cigarette smoke promotes a proinflammatory tumor microenvironment by stimulating the production of cytokines such as interleukin 6 (IL-6) and interleukin 8 (IL-8), both of which are involved in tumor progression, angiogenesis, and immune evasion (9–11). Another significant etiological factor in HNSCC, particularly in oropharyngeal cancers, is infection with human papillomavirus (HPV), most notably the high-risk genotype HPV-16, which contributes to carcinogenesis through distinct molecular mechanisms independent of standard risk factors (3, 12). Current standard treatment modalities for HNSCC typically involve surgical resection and/or platinum-based chemotherapy. Nevertheless, intrinsic or acquired resistance to therapy remains a major clinical challenge, resulting in a 5-year overall survival rate of approximately 50% (12, 13).

Therefore, alternative strategies for the treatment of HNSCC, including (neo) adjuvant therapies, are being studied. Ruthenium (Ru) complexes are considered a promising alternative to platinum-based anticancer drugs due to their similar properties and other benefits, such as increased treatment effect and lower toxicity *in vivo* (14, 15). They display numerous cytotoxic effects, such as induction of oxidative and endoplasmic reticulum stress (16). Ru complexes have also been studied in HNSCC tumors. Ru–arene complexes have demonstrated a cytotoxic effect in both HPV− and HPV+ HNSCC cell lines. The experimental results obtained with the Ru-based compound RuCy in 3D cell culture models demonstrated a promising cytotoxic effect on the tested cell lines (SCC-25, UPCI: SCC-154). In addition, RuCy exhibited an inhibitory effect on cell migration (17).

Functionalization of arene–Ru(II) complexes with therapeutically active bioligands offers further potential to enhance their antitumor efficacy (16). One such class of ligands is curcumin and its derivatives, which are well known for their broad spectrum of biological activities. Curcumin’s natural origin and multifunctional properties, particularly its anti-inflammatory, antitumoral, and antioxidant effects, make it a highly attractive candidate for incorporation into Ru(II)-based agents (18, 19). It should also be mentioned that more hydrophobic Ru(II)–arene complex ligands display significantly higher intracellular accumulation and efficiency than less hydrophobic ones (20). It is well known that curcumin and other curcuminoids display very low solubility (3, 19).

Furthermore, curcumin-based compounds have been extensively studied in the context of HNSCC, with numerous studies demonstrating their anticancer effects (3, 19, 21). The effect of curcumin is mainly mediated through the reduction of NF-κB expression and inhibition of its nuclear localization (22). Aberrant NF-κB activation plays a key role in HNSCC carcinogenesis (23, 24). Barnes et al. reported that 65% of clinical samples from head and neck cancer (HNC) patients display NF-κB (p65) expression. In these malignant tumors, the protein level was twice as high as in a benign tumor (25). Its overactivation is deeply associated with metastatic phenotype, drug resistance, and generally bad prognosis. For example, HNSCC patients with NF-κB overexpression display worse recurrence-free survival. Higher expression of NF-κB was also associated with reduced sensitivity to afatinib therapy (26). In addition, the expression levels of NF-κB-regulated gene products such as IL-6 and IL-8 (27, 28). Both IL-6 and IL-8 can induce epithelial-to-mesenchymal transition (EMT), stimulate angiogenesis and tumor growth, and promote tumor cell migration in oral squamous cell carcinoma (OSCC) (29, 30). It has also been studied that high levels of IL-6 are present in the tumor microenvironment of HNSCC, which is associated with poor prognosis and higher mortality in HNSCC patients (31, 32).

Ru–curcumin complexes show a promising cytotoxic effect on various types of cancer cells already at low concentrations. Li et al. studied Ru–polypyridyl complexes with curcumin on non-small cell lung (A549), breast (MCF-7), and gastric (SGF7901) tumor cell lines and found that they exhibited enhanced cytotoxicity compared to curcumin and cisplatin alone (33). Caruso et al. also studied Ru complexes on a number of tumor cell lines. Arene–Ru curcumioid complexes showed increased cytotoxicity on tumor cells, and this was probably due to the increased lipophilicity of curcuminoids (34).

However, for many of the benefits of Ru(II) complexes, the problem of a lack of selectivity and antitumor activity still persists. Therefore, we designed two structures (complexes 3 and 4) incorporating both Ru complexes and curcumin derivatives that combine the advantages of both curcumin and Ru complexes. The results show that complex 3 has a promising effect on both HPV− and HPV+ cell lines. It has a significant cytotoxic effect, as well as suppresses migration and cell invasion. In addition, it reduces the levels of important inflammatory mediators—NF-κB, IL-6, and IL-8. Compared to complex 3, complex 4 has a lower effect on all studied factors. Complex 4 is more effective on HPV− cell lines and more specifically on OSCC lines (such as CAL 27 and SCC-9). Complex 3 also has a significant immunomodulatory effect and suppresses NF-κB, IL-6, and IL-8. Taken together, these findings indicate that ruthenium-enhanced curcumin derivatives may serve as promising candidates for the development of multitarget agents with both anticancer and anti-inflammatory properties in the treatment of head and neck cancers.

## 2 Materials and methods

### 2.1 Materials

All chemicals were sourced from commercial suppliers and utilized directly without any additional purification steps. Nuclear Magnetic Resonance (NMR) spectra were acquired on a 400-MHz spectrometer (JEOL, Tokyo, Japan) at 25°C. The chemical shifts (δ) are given in parts per million (ppm), with coupling constants (J) measured in hertz (Hz). The ^1^H and ^13^C NMR shifts are referenced to TMS, and solvent signals from Dimethyl Sulfoxide (DMSO)-*d_6_* (2.50 ppm for ^1^H and 39.52 ppm for ^13^C) were used as references. Data analysis was performed using MestReNova software (version 14.2.1, Mestrelab Research S.L., Santiago de Compostela, SPAIN). Full assignment was performed using a combination of ^1^H, ^13^C, correlation spectroscopy (COSY), heteronuclear single quantum coherence (HSQC), and heteronuclear multiple bond correlation (HMBC) experiments. Mass spectrometry was conducted in positive ion mode using electrospray ionization (ESI^+^) with a high-resolution hybrid Ion Trap-Orbitrap mass spectrometer (LTQ Orbitrap Velos, Thermo Scientific, Waltham, Massachusetts, USA), **Supplementary Figures S21**, **S22**. Detailed NMR spectra for all synthesized products are available in **Supplementary Figures S12**-**S20**. The complexes were also characterized using Ultraviolet–Visible (UV–Vis) spectroscopy (**Supplementary Figure S21**), measured in a phosphate-buffered saline environment (PBS; the final PBS: DMSO ratio did not exceed 99:1). Characteristic absorption maxima were identified, and their dependence on concentration was evaluated. The linear dependence of absorbance on concentration within the given concentration range indicates that, under the measurement conditions, no significant aggregation, dissociation, or other interactions of complex 3 or 4 occur that would lead to spectral nonlinearity. Thus, it can be inferred that both complexes exhibit sufficient solubility in the buffered environment, which is crucial for their further application in biologically relevant experiments.

### 2.2 Preparation

#### 2.2.1 Preparation of compound 1

Tetrahydro-4H-pyran-4-one (0.60 g, 6 mmol) and quinoline-4-carbaldehyde (1.88 g, 12 mmol) were dissolved in ethanol (40 mL) and stirred at room temperature. LiOH·H_2_O (105 mg, 2.5 mmol) was then added to the reaction mixture. Stirring was continued overnight, and the reaction progress was monitored by Thin Layer Chromatography (TLC) using a Dichloromethane:Methanol (DCM: MeOH) (99:1, v/v) solvent system. Upon completion, water (200 mL) was added to the reaction mixture, resulting in the precipitation of the product. The solid was collected by filtration, washed thoroughly with EtOH, and dried to yield the desired compound 1 as a pale-yellow solid (2.05 g, 90%). ^1^H NMR (400 MHz, DMSO-*d_6_*) δ: 8.98 (d, *J* = 4.3 Hz, 2H), 8.29 (s, 2H), 8.12 (d, *J* = 8.4 Hz, 2H), 8.08 (d, *J* = 6.9 Hz, 2H), 7.86 (t, *J* = 7.6 Hz, 2H), 7.73 (t, *J* = 7.6 Hz, 2H), 7.40 (d, *J* = 4.4 Hz, 2H), 4.80 (s, 4H). ^13^C NMR (101 MHz, DMSO-*d_6_*) δ: 184.28, 150.15, 147.79, 139.08, 137.46, 130.77, 129.93, 129.76, 127.47, 125.79, 124.40, 121.33, 67.57.

#### 2.2.2 Preparation of compound 2

Cyclopentanone (252 mg, 3 mmol) and nicotinaldehyde (643 mg, 6 mmol) were dissolved in ethanol (50 mL) and stirred at room temperature. LiOH·H_2_O (40 mg, 1 mmol) was then added to the reaction mixture. Stirring was continued overnight, and the reaction progress was monitored by TLC using a DCM: MeOH (99:1, v/v) solvent system. Upon completion, water (100 mL) was added to the reaction mixture, resulting in the precipitation of the product. The solid was collected by filtration, washed thoroughly with water, and dried to yield the desired compound 2 as a pale-yellow solid (566 mg, 72%). ^1^H NMR (400 MHz, DMSO-*d_6_*) δ: 8.89 (d, *J* = 2.2 Hz, 2H), 8.60 (dd, *J* = 4.7, 1.6 Hz, 2H), 8.11 (dt, *J* = 8.0, 2.0 Hz, 2H), 7.55–7.46 (m, 4H) 3.15 (s, 4H). ^13^C NMR (101 MHz, DMSO-*d_6_*) δ: 194.86, 151.72, 149.86, 139.59, 136.97, 131.16, 129.47, 123.93, 25.89.

#### 2.2.3 Preparation of Ru complex 3

Dichloro(*p*-cymene)ruthenium dimer (32 mg, 0.05 mmol) and compound 1 (20 mg, 0.05 mmol) were dissolved in 20 mL of chloroform, and the mixture was refluxed for 24 h. Upon completion, all starting materials were consumed, and a solid precipitate had formed. The solvent was removed under reduced pressure, and the crude product was recrystallized from a mixture of hexane and dichloromethane to afford complex 3 as a dark green solid (45 mg, 86% yield). ^1^H NMR (400 MHz, DMSO-*d_6_*) δ: 8.98 (d, *J* = 4.4 Hz, 2H, H2), 8.29 (s, 2H, H11), 8.11 (d, *J* = 7.2 Hz, 2H, H9), 8.06 (d, *J* = 7.0 Hz, 2H, H6), 7.86 (t, *J* = 7.8 Hz, 2H, H8), 7.72 (t, *J* = 7.8 Hz, 2H, H7), 7.40 (d, *J* = 4.6 Hz, 2H, H3), 5.81 (d, *J* = 6.5 Hz, 4H, H18), 5.77 (d, *J* = 6.5 Hz, 4H, H17), 4.79 (s, 4H, H14), 2.83 (hept, *J* = 6.9 Hz, 2H, H20), 2.08 (s, 6H, H15), 1.19 (d, *J* = 7.0 Hz, 12H, H21).^13^C NMR (101 MHz, DMSO-*d_6_*) δ: 184.3 (C13), 150.1 (C2), 147.8 (C10), 139.1 (C4), 137.5 (C12), 130.8 (C11), 129.9 (C8), 129.7 (C9), 127.5 (C7), 125.8 (C5), 124.4 (C6), 121.3 (C3), 106.3 (C19), 100.1 (C16), 86.3 (C17), 85.5 (C18), 67.6 (C14), 29.9 (C20), 21.5 (C21), 17.8 (C15). High-Resolution Mass Spectrometry (HRMS) (ESI^+^) calcd for [M − (Ru(*p*-cymene)Cl_2_)] *m*/*z* = 685.0957 (C_35_H_33_Cl_2_N_2_O_2_Ru), found *m*/*z* = 685.0956.

#### 2.2.4 Preparation of Ru complex 4

Dichloro(*p*-cymene)ruthenium dimer (46 mg, 0.07 mmol) and compound 2 (20 mg, 0.071 equiv.) were combined in 20 mL of chloroform, and the reaction mixture was refluxed for 24 h. Upon completion, the starting materials were fully consumed, and a solid was observed in the reaction mixture. The solvent was removed under reduced pressure, and the resulting residue was recrystallized from a hexane/dichloromethane mixture to yield complex 4 as a yellow solid (39 mg, 60% yield). ^1^H NMR (400 MHz, DMSO-*d*_6_) δ: 8.90 (d, *J* = 2.3 Hz, 2H, H2), 8.60 (dd, *J* = 4.8, 1.6 Hz, 2H, H6), 8.11 (d, *J* = 6.1 Hz, 2H, H4), 7.52 (dd, *J =* 4.8, 2.0 Hz, 2H, H5), 7.50 (s, 2H, H7),5.82 (d, *J* = 6.2 Hz, 4H, H14), 5.78 (d, *J* = 6.2 Hz, 4H, H13), 3.15 (s, 4H, H10), 2.83 (h, *J* = 7.0 Hz, 2H, H16), 2.08 (s, 6H, H11), 1.19 (d, *J* = 6.9 Hz, 13H, H17). ^13^C NMR (101 MHz, DMSO-*d*_6_) δ: 194.9 (C9), 151.8 (C2), 149.9 (C6), 139.6 (C8), 137.0 (C4), 131.2 (C3), 129.5 (C7), 123.9 (C5), 106.3 (C15), 100.1 (C12), 86.4 (C13), 85.5 (C14), 30.0 (C16), 25.9 (C10), 21.5 (C17), 17.9 (C11). HRMS (ESI^+^) calcd for [M − (Ru(*p*-cymene)Cl_3_)] *m*/*z* = 533.0928 (C_27_H_28_ClN_2_ORu), found *m*/*z* = 533.0929.

### 2.3 Cell lines

HGF, SCC-9, and FaDu cells were cultured in DMEM: F12 (Gibco, Inchinnan, Scotland, UK), while CAL 27 and TR146 cells were maintained in DMEM and Ham’s F12, respectively. Detroit 562 and THP1-Blue™ NF-κB reporter monocytes were cultured in Roswell Park Memorial Institute (RPMI)-1640, and Hep-2 and KB cells were cultured in EMEM (all cell culture media were obtained from Gibco, UK). All cultures were supplemented with 10% (v/v) fetal bovine serum (FBS, qualified, US origin; Gibco), except for HGF, which was supplemented with 5% FBS, and THP1-Blue cells, which were supplemented with heat-inactivated FBS. All cell lines were supplemented with 1% (v/v) penicillin–streptomycin (Gibco, UK); SCC-9 cultures additionally contained hydrocortisone (Sigma-Aldrich, Merck, Darmstadt, Germany), and THP1-Blue cells were supplemented with 100 μg/mL Normocin (InvivoGen, San Diego, California, USA). Cells were incubated at 37°C in a humidified atmosphere containing 5% CO_2_. For more detailed information about the cell lines, see the **Supplementary Materials**.

### 2.4 Molecular docking studies of NF-κB, IL-6, IL-8, and IL-6R with complexes 3 and 4

The 3D crystal structures of the target proteins (IL-6, IL-6R, IL-8, and NF-κB) were obtained from the RSCB Protein Data Bank with PDB IDs 1P9M (for the crystal structure of the human IL-6/IL-6R complex), 1ALU (human IL-6 monomer), 3IL8 (human IL-8 dimer), and 1NFI (human NF-κB:IκB complex) (35), and biologically relevant oligomeric assemblies (dimers or higher-order multimers) were constructed where necessary. Similarly, the coordinates of a p50–p65 NF-κB dimer were obtained by removing the coordinates corresponding to IκB using COOT (36).

The retrieved structures underwent a preparation process prior to docking. This process involved the removal of all bound water molecules and ligands, in accordance with protocols from AutoDock Vina software (37). In the structures obtained from the PDB database, all bound water molecules and ligands were removed and saved as PDB files. These edits were carried out in UCSF ChimeraX (38). The 3D structures of tested compounds (complexes 3 and 4) were generated using 3D ChemDraw software. All structural models were energy-minimized using YASARA (39).

Molecular docking was carried out using AutoDock Vina software. The resulting receptor–ligand complexes were visualized and analyzed using PyMOL, UCSF ChimeraX, and BIOVIA Discovery Studio Visualizer. Interaction diagrams of the protein–ligand complexes were generated with BIOVIA DSV, while 3D representations of the docked complexes were produced in UCSF ChimeraX (38, 40, 41).

The AutoDock Vina suite cannot model the η^6^–arene coordination of ruthenium; therefore, we adopted a two‐step, “sequential” docking strategy. In the first step, we docked an organic fragment lacking the benzyl ring system to the target protein (with ruthenium substituted by phosphorus for the calculations). We then treated the resulting protein–fragment complex as a rigid receptor and performed a focused docking of the benzyl‐ring fragment to rebuild the full complex ligand. Finally, the binding free energies of the complete complex (3/4)–protein complexes were calculated using the PRODIGY server (42).

### 2.5 MTT viability assay

A colorimetric 3-(4,5-dimethylthiazolyl)-2,5-diphenyltetrazolium bromide (MTT) cell metabolic activity assay was used to assess the cytotoxicity of complexes 3 and 4. The cells (described above) were cultured under standard conditions. Cells were seeded in 96-well plates at a density of 10,000 cells per well (200 μL) and allowed to grow for 24 h. The medium was then replaced with medium containing complex 3 or 4 at a concentration ranging from 0 to 200 μM, and the cells were incubated for an additional 24, 48, and 72 h. Subsequently, the medium was replaced with MTT solution, consisting of yellow tetrazolium dye 3-(4,5-dimethylthiazol-2-yl)-2,5-diphenyltetrazolium bromide (Sigma-Aldrich, Merck, Germany), and incubated for 2 h at 37°C. The dye was then removed, and the resulting purple formazan formed in viable cells was dissolved in DMSO, followed by shaking for 30 s. Absorbance was measured at 570 nm using an INFINITE 200 PRO microplate reader (TECAN, Männedorf, Switzerland). All experiments were performed in quadruplicate and repeated three times.

The cell inhibitory concentration (IC) was calculated using the equation IC = (A_complex_**_3_**_/complex_**_4_** well/mean A_control_ wells) × 100%. The half-maximal inhibitory concentration (IC_50_) was determined from dose–response curves using GraphPad Prism 10 (GraphPad Software Inc., La Jolla, CA, USA). The selectivity index toward cancer cells was calculated as a ratio of the average IC_50_ value for HGF to the IC_50_ value for the corresponding cancer cell line.

### 2.6 Colony formation assay

Cells were seeded in six-well plates at a density of 100,000 cells per well (10,000 cells/200 μL) and grown for 24 h. The medium was then replaced with medium containing complex 3 or 4 at the IC_50_ concentration and cultured for an additional 24 h. Subsequently, cells were reseeded in new six-well plates at a density of 500 cells per well and grown for approximately 2 weeks. The cell culture medium was replaced every 2–3 days. When visible colonies appeared in the six-well plates, the culture was terminated, and the cells were washed twice with ice-cold PBS. The cells were then fixed with ice-cold methanol for 10 min and stained with 0.5% crystal violet solution for 30 min at room temperature. Experiments were performed in four independent replicates. The results were analyzed using the ImageJ plugin ColonyArea, which is optimized for standard analysis of colony formation assays. The plugin processes each well individually and determines not the number of colonies, but the area of the well covered by cells, also considering staining intensity.

### 2.7 Wound healing assay

The wound healing activity of complexes 3 and 4 was evaluated using the ibidi Culture-Insert 2 Well in a μ-dish. Cells were plated by applying 70 μL of cell suspension at a density of 50,000 cells into both wells of the Culture-Insert 2 Well and grown over the weekend. Once confluency was achieved, sterile tweezers were used to detach the Culture-Insert 2 Well from the μ-dish. The cell layer was then gently washed with PBS. Subsequently, the cell layer was treated with medium containing complex 3 or 4 at the IC_25_ concentration, and wound closure was observed and photographed using an inverted light microscope (0–55 h of incubation). Experiments were performed in four independent replicates.

### 2.8 NF-κB activity assay + MTT viability assay

THP1-Blue NF-κB cells, NF-κB secreted embryonic alkaline phosphatase (SEAP) reporter monocytes (InvivoGen, USA), were assayed after 20–24 h of incubation following the manufacturer’s protocol. Briefly, cells were seeded at 1 × 10^6^ cells/mL in RPMI medium in 96-well plates (180 μL) and treated with complex 3 or 4, Lipopolysaccharide (LPS) (10 ng/mL; LPS-EB, O111:B4, InvivoGen, USA), or a combination of both, and incubated overnight at 37°C with 5% CO_2_ in a total volume of 200 μL. The next day, NF-κB activation was assessed via the secretion of SEAP. Supernatants (20 μL) were transferred to 96-well plates, and 180 μL of Quanti-Blue was added. Plates were incubated for 2 h at 37°C, and absorbance was measured at 600 nm using an INFINITE 200 PRO microplate reader (TECAN, Switzerland).

A total of 20 μL of sterile filtered MTT (Sigma-Aldrich, Merck, Germany) solution (5 mg/mL in PBS) was added to the remaining overnight culture of THP1-Blue NF-κB cells from the above NF-κB activity assay in 96-well plates, which were incubated at 37°C (see above). After 2 h of incubation at 37°C, the supernatant was removed, and the purple formazan product generated in the cells was dissolved by the addition of 100 μL of DMSO to each well. The plates were then gently shaken for 30 s at room temperature to dissolve the precipitates. The absorbance was measured at 570 nm using an INFINITE 200 PRO microplate reader (TECAN, Switzerland) (43).

### 2.9 Determination of intracellular p65-NF-κB

To determine NF-κB p65 transcription factor levels by enzyme-linked immunosorbent assay (ELISA), an assay kit was used (Abcam, ab133112, Cambridge, UK). After treatment of THP1-Blue NF-κB cells with complex 3, complex 4, curcumin, and LPS (10 ng/mL), or with LPS alone (10 ng/mL), cells were lysed with RIPA Lysis and Extraction (Thermo Scientific) and centrifuged at 1,500×*g* for 10 min. The supernatants were collected and used for the determination of intracellular p65-NF-κB by ELISA.

In this assay, a specific double-stranded DNA (dsDNA) sequence containing the NF-κB p65 response element is immobilized onto the wells of a 96-well plate. NF-κB p65 present in the sample binds specifically to these response elements. Bound p65 is subsequently detected using a primary antibody specific for NF-κB p65, followed by a horseradish peroxidase (HRP)-conjugated secondary antibody. The relevant background values were subtracted from the measured values. Absorbance was measured at 450 nm using an INFINITE 200 PRO microplate reader (Tecan, Switzerland).

### 2.10 IKKβ inhibition assay

IκB kinase beta (IKKβ) inhibition was determined using the IKKβ Kinase Assay Kit (BPS Bioscience, San Diego, California, USA). IKKβ activity was measured in the presence of complex 3 (1, 3, and 8 μM), complex 4 (1, 5, and 15 μM), or curcumin (5, 10, and 20 μM) according to the manufacturer’s protocol. Kinase-Glo^®^ MAX (Promega, #V6071, Madison, Wisconsin, USA) was used as the detection reagent, with an incubation time of 15 min. Blank values were subtracted from all measurements. Luminescence was measured with an integration time of 1 s using a Spark Microplate Reader (Tecan, Switzerland).

### 2.11 ELISA assay (IL-6/IL-6R/IL-8)

Human ELISA kits for IL-6, IL-6R, and IL-8 (Sigma-Aldrich, Merck, Germany) were used to quantify cytokine levels after treatment with complex 3 or 4. Briefly, 96-well plates precoated with specific antibodies were used to capture IL-6, IL-6R, and IL-8 from samples. Standards and samples (containing complex 3, complex 4, or curcumin) were added to the wells. Each sample was supplemented with 500 pg/mL of IL-6/IL-6R/IL-8. Positive control wells contained only the IL-6/IL-6R/IL-8 standard (PeproTech, Thermo Fisher Scientific, Waltham, Massachusetts, USA). Samples were treated with complex 3 (1, 3, and 8 μM), complex 4 (1, 5, and 15 μM), or curcumin (5, 10, and 20 μM). After incubation, wells were washed, and biotinylated antihuman antibodies were added, followed by washing and addition of HRP-conjugated streptavidin. Finally, the TMB substrate was added to develop a color proportional to the cytokine concentration, and the reaction was stopped with the stop solution. Absorbance was measured at 450 nm using an INFINITE 200 PRO microplate reader (TECAN, Switzerland).

### 2.12 Statistical analysis

The graphs of colony formation assay (CFA), MA, NF-κB, IL-6, IL-6R, and IL-8 activity are presented as the mean ± SEM from at least three independent experiments. Differences in these assays were assessed using one-way ANOVA with Dunnett’s multiple comparison tests. All data were analyzed using GraphPad Prism 10 (GraphPad Software Inc., La Jolla, CA, USA). Additionally, *p*-values less than 0.05 were considered to be statistically significant (not significant (ns), ^*^p < 0.05, ^**^p < 0.01, ^***^p < 0.001, and ^****^p < 0.0001).

## 3 Results

### 3.1 Synthesis of ruthenium–curcumin complex

**Scheme 1:**
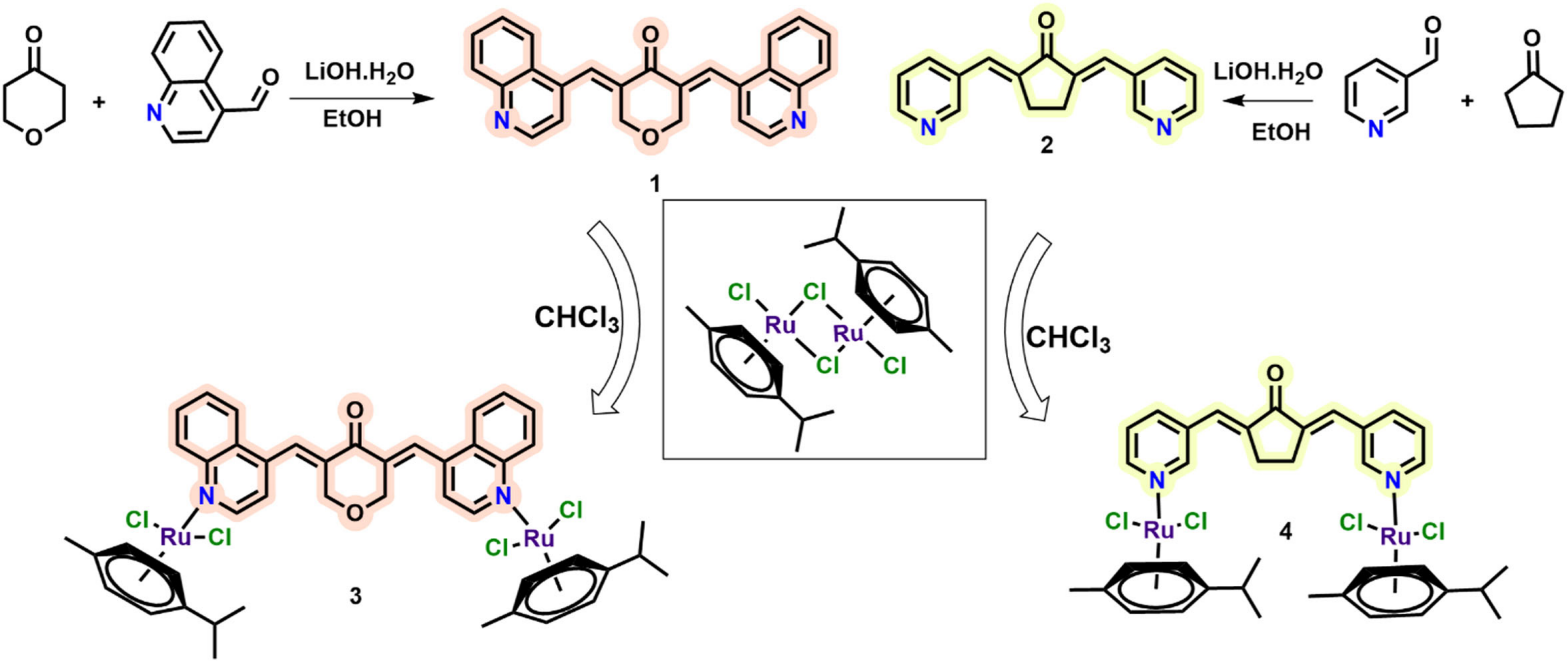
Preparation of curcumin–ruthenium complex.

The bidentate ligands were synthesized via a Claisen–Schmidt condensation between an appropriate aldehyde and ketone, as shown in **Scheme 1**. Compound 1 was obtained in excellent yield (90%), and compound 2 was prepared in 72% yield. The precipitation of compound 1 was carried out in ethanol, whereas compound 2 was precipitated from water. The structures of both ligands corresponded to published data (44, 45). The synthesized bidentate ligands were then reacted with dichloro(*p*-cymene)ruthenium(II) dimer in a 1:1 molar ratio under reflux for 24 h. The resulting ruthenium complexes were purified by recrystallization using hexane and dichloromethane, affording complex 3 as a dark green powder (86%) and complex 4 as a yellow solid (60%).

Both complexes were characterized by HRMS and NMR spectroscopy. During HRMS analysis, the complexes tend to lose one side of the bidentate ligand–ruthenium coordination, resulting in fragmentation. However, NMR spectroscopy confirms that the complexes are symmetric, with the bidentate ligand coordinating ruthenium centers on both sides. The spectra show two aromatic doublets (integrating for eight protons) in the range of δ 5.77–5.88 ppm, corresponding to the *p*-cymene ring protons, consistent with symmetrical structures. The isopropyl group of the *p*-cymene ligand displays characteristic signals: a doublet around δ 1.18 ppm for the methyl protons (CH_3_) and a septet at δ 2.8 ppm for the methine proton (CH in CH(CH_3_)_2_). An additional singlet around δ 2.08 ppm was attributed to the methyl group on the *p*-cymene ring. In the curcuminoid framework, the aromatic proton resonances appeared in the region of δ 7.39–8.98 ppm for both complexes. The methylene (–CH_2_–) protons of the central bridge, due to the overall symmetry of the complexes, appeared as a singlet at δ 4.48 ppm for complex 3 and δ 3.15 ppm for complex 4 (for detailed NMR assignments, see the ESI).

### 3.2 Molecular docking studies of NF-κB, IL-6, IL-8, and IL-6R with complexes 3 and 4

Binding energy calculations were performed for complexes 3 and 4, their precursors (compounds 1 and 2), and native curcumin against NF-κB, IL-6, IL-8, and IL-6R. Both complexes 3 and 4 exhibited significant affinity for NF-κB, IL-6, and IL-8, with the highest binding affinities observed for NF-κB and IL-6. Notably, complex 4 displayed binding energies over − 10 kcal/mol for both targets. They also exhibited affinity for IL-6R; however, the binding affinity of complex 4 toward IL-6R (7.68 kcal/mol) was substantially lower than its binding affinity toward IL-6d (− 11.69 kcal/mol). In comparison, native curcumin exhibited lower binding affinity for NF-κB and IL-8 targets (− 5.36 and − 5.44 kcal/mol, respectively), whereas its affinity for IL-6 (− 8.62 kcal/mol) was comparable to that of complex 3, suggesting a potentially conserved mode of interaction with this cytokine. Overall, complex 4 demonstrated the strongest binding to IL-6, which may underlie its enhanced anti-inflammatory and antitumor potential relative to native curcumin (**Table 1**). Docking poses of native curcumin with IL-6, IL-8, and NF-κB are shown in **Supplementary Figures S1**-**S3**.

**Table 1:** Results of predicted binding energies of complex 3, complex 4, compound 1, compound 2, and curcumin with targets (NF-κB, IL-6d, and IL-8d).

Receptor	Binding energy (kcal/mol)				
	Complex 3	Compound 1	Complex 4	Compound 2	Curcumin
NF-κB	− 7.75	− 7.75	− 10.50	− 7.30	− 5.36
IL-6d	− 8.91	− 8.66	− 11.69	− 6.49	− 8.62
IL-8d	− 7.49	− 6.85	− 7.70	− 7.17	− 5.44

Calculations of binding energies were also performed on compounds in which the ruthenium atom and the molecules coordinated to it were removed. This approach allowed us to determine the effect of ruthenium on the binding ability of the compounds (complexes 3 and 4). The results indicate that the presence of ruthenium influences the binding ability of the complexes. In complex 3, ruthenium contributes to an increase in binding energy, particularly for the IL-6d and IL-8 targets. In contrast, complex 4 shows an increase in binding energy across all targets. The largest change in binding energy upon removal of ruthenium is observed for complex 4 relative to IL-6d; complex 4 containing ruthenium has a binding energy of 11.69 kcal/mol, whereas compound 2 shows only 6.49 kcal/mol.

**Figure 1:**
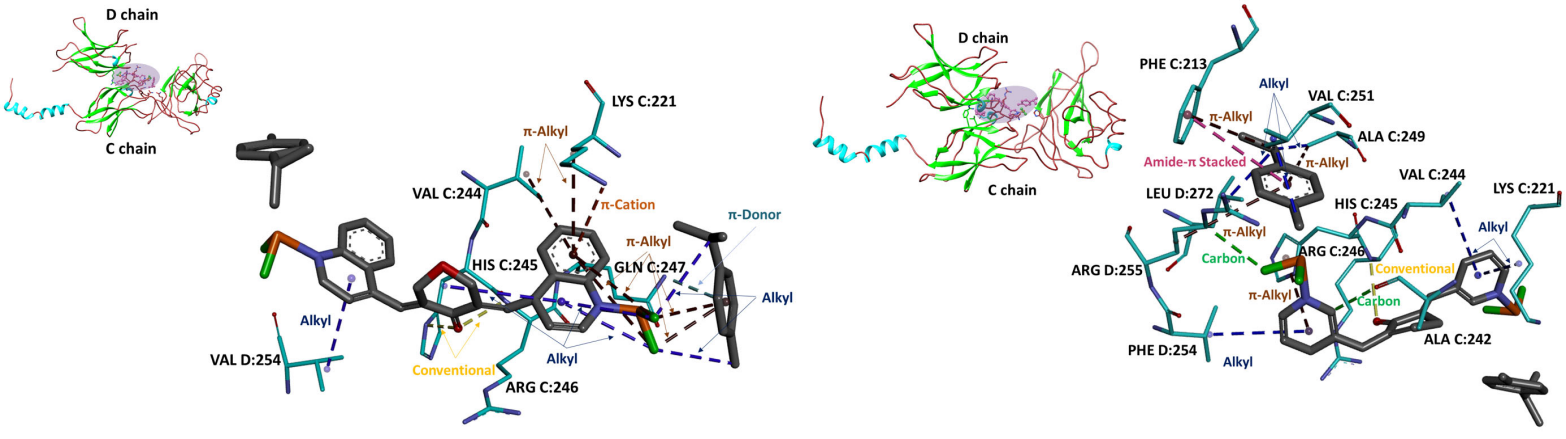
Docking poses of complex 3 (on the left) and complex 4 (on the right) (colored magenta in the violet area) in the NF-κB (binding energy: − 7.75 and − 10.50 kcal/mol, respectively). The upper-left inset provides a global view of the NF-κB in cartoon representation (cyan helices, brown coils, and green strands).

**Figure 1** illustrates the placement of the complex 3 (on the left) and complex 4 (on the right) as ligands on the surface of NF-κB. In the case of complex 3, one methyl isopropylbenzene ring forms two *alkyl* and two π-*alkyl* contacts with the ligand’s own quinoline moiety, and also establishes a π-*donor* interaction with GLN C:247. The benzene ring of the quinoline moiety engages in *π-alkyl* contacts with LYS C:221 and VAL C:244 and, with the same ring, creates *a π-cation* interaction with the positively charged LYS C:221. An additional alkyl interaction is observed between the pyridine ring of the quinoline moiety and the tetrahydropyranone ring. The carbonyl oxygen of that tetrahydropyranone ring forms two *conventional hydrogen* bonds with HIS C:245 and ARG C:246. Finally, the pyridine ring of the distal quinoline system forms an alkyl interaction with VAL D:254.

In the complex 4 binding pose, ARG C:246 forms a *conventional hydrogen* bond with an oxygen atom from the cyclopentanone ring, while ARG D:255 forms a *halogen–hydrogen* bond with a chloride atom from the ruthenium moiety. ALA C:242 forms a weaker *carbon–hydrogen* bond with a pyridine ring*. Alkyl* interactions occur between VAL C:251, ALA C:249, and LEU D:272 with the methyl isopropylbenzene group, as well as between PHE D:254 and a pyridine ring, and between VAL C:244 and LYS C:221 with another pyridine ring. Additionally, the methyl isopropylbenzene group forms *π–alkyl* interactions with ARG D:255, ALA C:249, and PHE C:213, while HIS C:245 interacts with a pyridine ring of the ligand via *π–alkyl* contact. Furthermore, PHE C:213 also participates in an *amide–π stacking* interaction with the methyl isopropylbenzene portion of the ligand.

**Figure 2:**
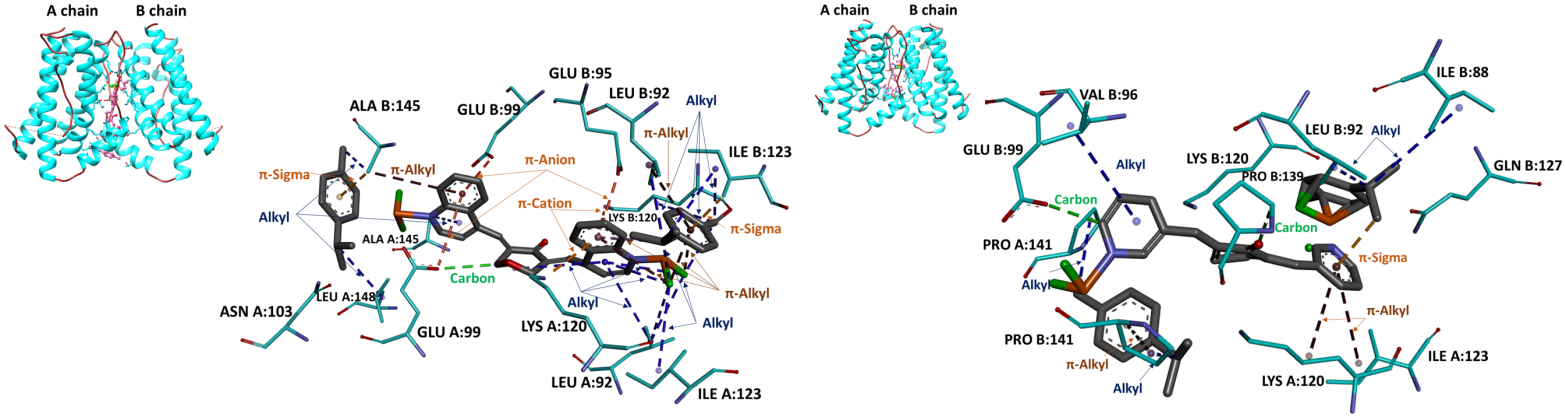
Docking poses of complex 3 (on the left) and complex 4 (on the right) (colored magenta) in the dimeric form of IL-6 (binding energy: − 8.91 and − 11.69 kcal/mol, respectively). The upper-left inset provides a global view of the IL-6 dimer in cartoon representation (cyan helices and brown coils).

**Figure 2** illustrates the docking of complex 3 (on the left) and complex 4 (on the right) as ligands on the surface of the IL-6 dimer. In the case of complex 3, stabilization is achieved through multiple interactions. A week *carbon–hydrogen* bond is formed between GLU A:99 and the tetrahydropyranone ring. One methyl isopropylbenzene ring establishes two *alkyl* interactions with ALA B:145 and LEU A:148, and additionally engages ALA B:145 in a *π–sigma* contact, while its nearby pyridine ring of the quinoline moiety forms an additional *alkyl* interaction with ALA A:145. The second methyl isopropylbenzene ring participates in a *π–sigma* interaction with ILE B:123, *π–alkyl* and *alkyl* contacts with LEU B:92 and ILE B:123, and *alkyl* interactions with LEU B:92, ILE B:123, as well as a *π–alkyl* contact with the ligand’s own quinoline core. The quinoline moiety itself forms *alkyl* interactions with ILE A:123 and LEU A:92 and an intramolecular *π–alkyl* contact with the tetrahydropyranone ring. Its benzene ring additionally engages in *π–cation* interactions with LYS A:120 and LYS B:120, and *π–anion* contacts with GLU B:99, GLU A:99, and GLU B:95.

In the complex 4 binding pose, the ligand is primarily stabilized by a network of hydrophobic interactions. These include *alkyl* contacts between VAL B:96 and the pyridine ring, as well as interactions involving LEU B:92 and PRO B:141 with one methyl isopropylbenzene, and additional *alkyl* contacts between LEU B:92 and ILE B:88 with a second methyl isopropylbenzene. The pyridine ring further engages *π–alkyl* interactions with ILE A:123 and LYS A:120, forming the protein’s hydrophobic pocket. Notably, a *π–sigma* interaction occurs within the ligand between the pyridine ring and a neighboring methyl group of the methyl isopropylbenzene moiety. Additionally, carbon–hydrogen bonds are formed between GLU B:99 and the pyridine group, and between PRO B:139 and an oxygen atom of the cyclopentanone ring.

**Figure 3:**
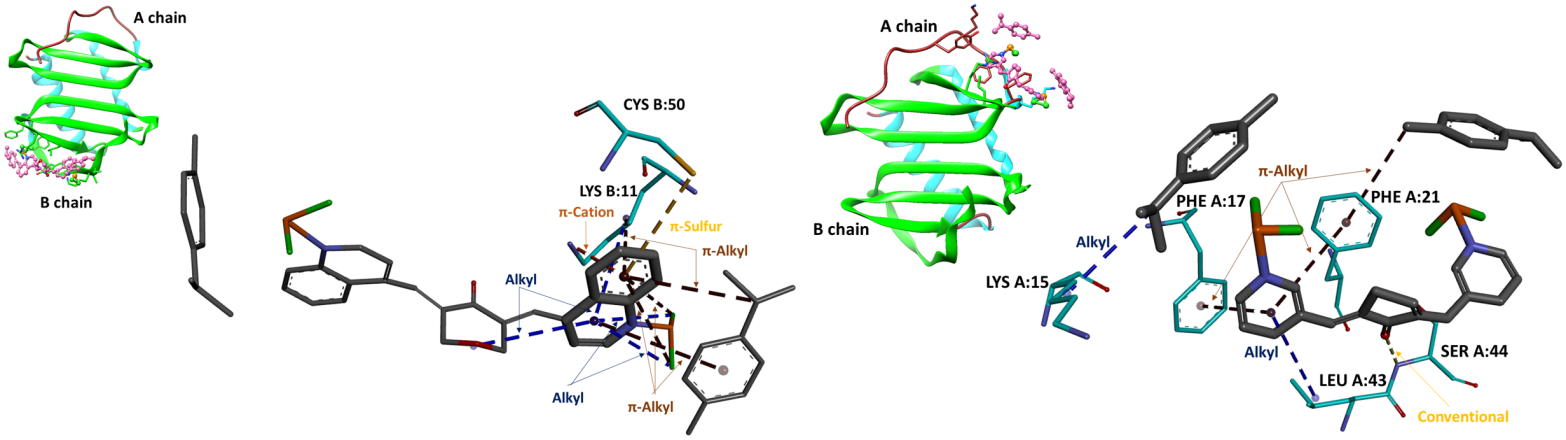
Docking poses of complex 3 (on the left) and complex 4 (on the right) (colored magenta) in the dimeric form of IL-8 (binding energy: − 7.49 and − 7.70 kcal/mol, respectively). The upper-left inset provides a global view of the IL-8 in cartoon representation (cyan helices, brown coils, and green strands).

**Figure 3** illustrates the placement of complex 3 (on the left) and complex 4 (on the right) as ligands on the surface of the IL-8 dimer. In the structure of complex 3, one methyl isopropylbenzene ring forms an *alkyl* contact with LYS A:15, while the pyridine ring makes an additional *alkyl* contact with LEU A:43. This pyridine ring also engages in *π*–*alkyl* interaction with PHE A:17 and PHE A:21; a further *π*–*alkyl* bond is observed between PHE A:21 and the methyl group of the methyl isopropylbenzene ring. Finally, a *conventional hydrogen* bond links the carbonyl oxygen of the cyclopentanone ring to SER A:44.

Similarly, in complex 4, one methyl isopropylbenzene ring forms five *π*–*alkyl* contacts with the ligand’s own quinoline moiety. The benzene ring of the quinoline moiety engages LYS B:11 through a *π–cation* interaction and an additional *π–alkyl* contact, while simultaneously forming a *π–sulfur* interaction with CYS B:50. The pyridine ring of the same quinoline moiety forms one *alkyl* contact with LYS B:11, two *alkyl* interactions with the ruthenium area, and another *alkyl* contact with the ligand’s tetrahydropyranone ring.

The studied compounds, particularly complexes 3 and 4, exhibit significant affinity (as indicated by binding energy) for tested protein targets, including NF-κB and, notably, the cytokines IL-6d and IL-8d. These results suggest that complexes 3 and 4 may act as potent inhibitors of NF-κB, IL-6, and IL-8 signaling.

### 3.3 Cytotoxicity assay

**Figure 4:**
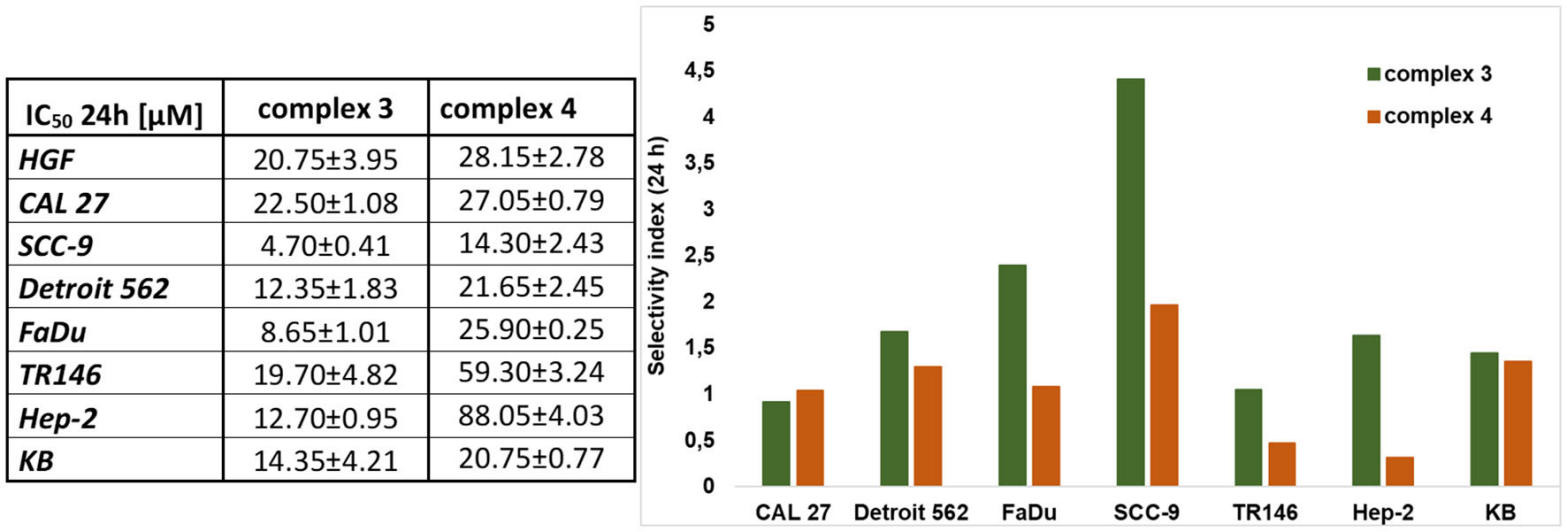
Cytotoxic effect and selectivity index of complexes 3 and 4. The MTT viable count assay was used to determine the effects. The IC_50_ values for each cell line (HGF, CAL 27, SCC-9, Detroit 562, FaDu, TR146, Hep-2, and KB) are shown in the table (left). The selectivity index (right) is determined for each cancer cell line against the healthy HGF cell line and was analyzed using the same assay. Data are presented as the mean ± SD of three independent experiments (*n* = 3).

The cytotoxic effects of complexes 3 and 4 were evaluated in normal (HFG) and tumor (CAL 27, SCC-9, Detroit 562, FaDu, TR146, Hep-2, and KB) cells using the standard MTT viability assay. Cells were exposed to complex 3 or 4 at concentrations ranging from 0 to 200 μM under standard conditions in complete cell culture medium, and cell viability was measured after 24, 48, and 72 h of treatment. The IC_50_ values after 24 h and a graphical representation of the selectivity index are shown in **Figure 4**. The IC_50_ values after 48 and 72 h are provided in **Supplementary Table S2**, and the corresponding graphical curves for 24, 48, and 72 h are shown in **Supplementary Figure S5**.

Complex 3 is more cytotoxic compared to complex 4. Both complexes showed the highest selectivity for the SCC-9 (OSCC—isolated from the tongue tissue) cell line, with IC_50_ values of 4.70 μM for complex 3 and 14.30 μM for complex 4. Complex 3 also exhibited selectivity for FaDu cell lines, with a cytotoxic effect observed at a concentration as low as 8.65 μM. Additionally, complex 3 demonstrated a relatively strong cytotoxic effect on the HPV+ lines Hep-2 and KB, with IC_50_ values of 12.70 and 14.35 μM, respectively. Complex 4 showed a cytotoxic effect only at concentrations above 20 μM in all other tested lines (except SCC-9).

Numerous studies have investigated the cytotoxic effects of native curcumin on HNC cell lines. For example, in the CAL 27 cell line, curcumin demonstrated statistically significant cytotoxicity at concentrations around 50 μM, with a reported IC_50_ value of 49.90 μM (46, 47). Similarly, for the SCC-9 cell line, cytotoxic effects were observed at approximately 40 μM, with an IC_50_ of 40.9 μM (48, 49). In the FaDu cell line, the IC_50_ was reported to be 24.8 μM (48). In the Detroit 562 cell line, curcumin showed cytotoxicity at concentrations around 20 μM (50, 51). For the HPV-positive Hep-2 cell line, the IC_50_ value was approximately 50 μM (52), while in the KB cell line, curcumin exhibited an IC_50_ of 8.84 μg/mL after 48 h of treatment (53). Importantly, in the noncancerous control cell line HGF, curcumin did not significantly affect cell viability within the concentration range of 0.1–20 μM (54).

### 3.4 Cell proliferation and migration

**Figure 5:**
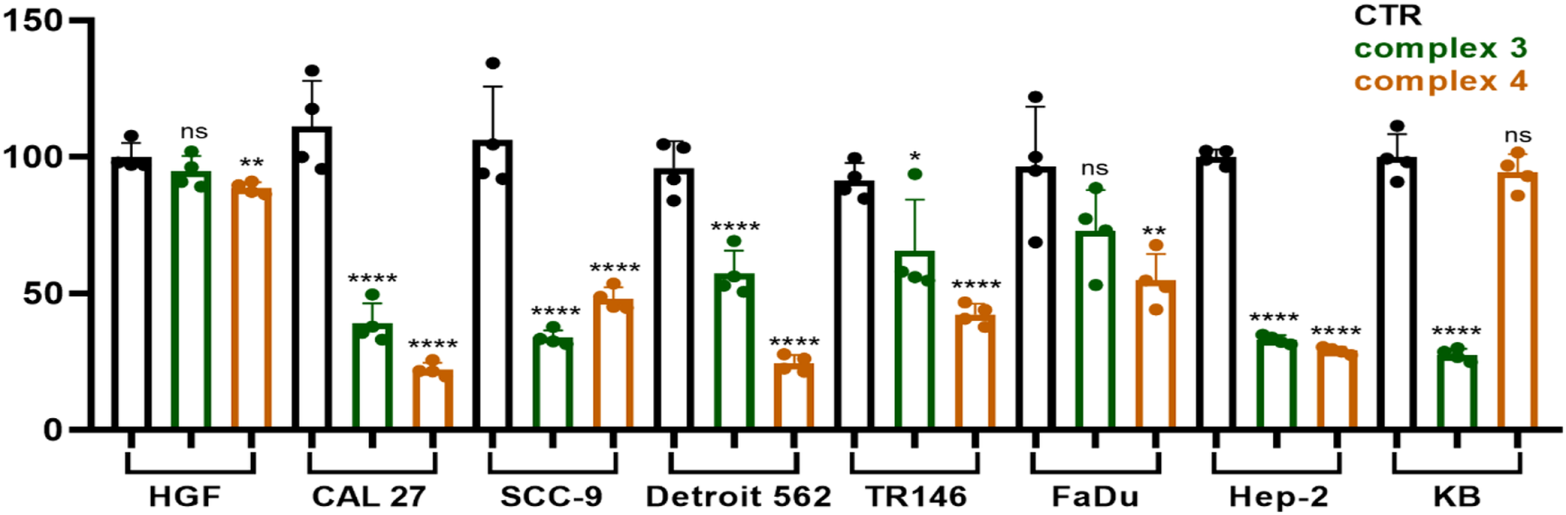
CFA was used to determine the cloning formation rate of treated cells compared with control (nontreated cells). Statistical analysis was performed using a one-way ANOVA with Dunnett’s multiple comparison tests. Results are reported as not significant (ns), ^*^p < 0.05, ^**^p < 0.01, and ^****^p < 0.0001.

**Figure 6:**
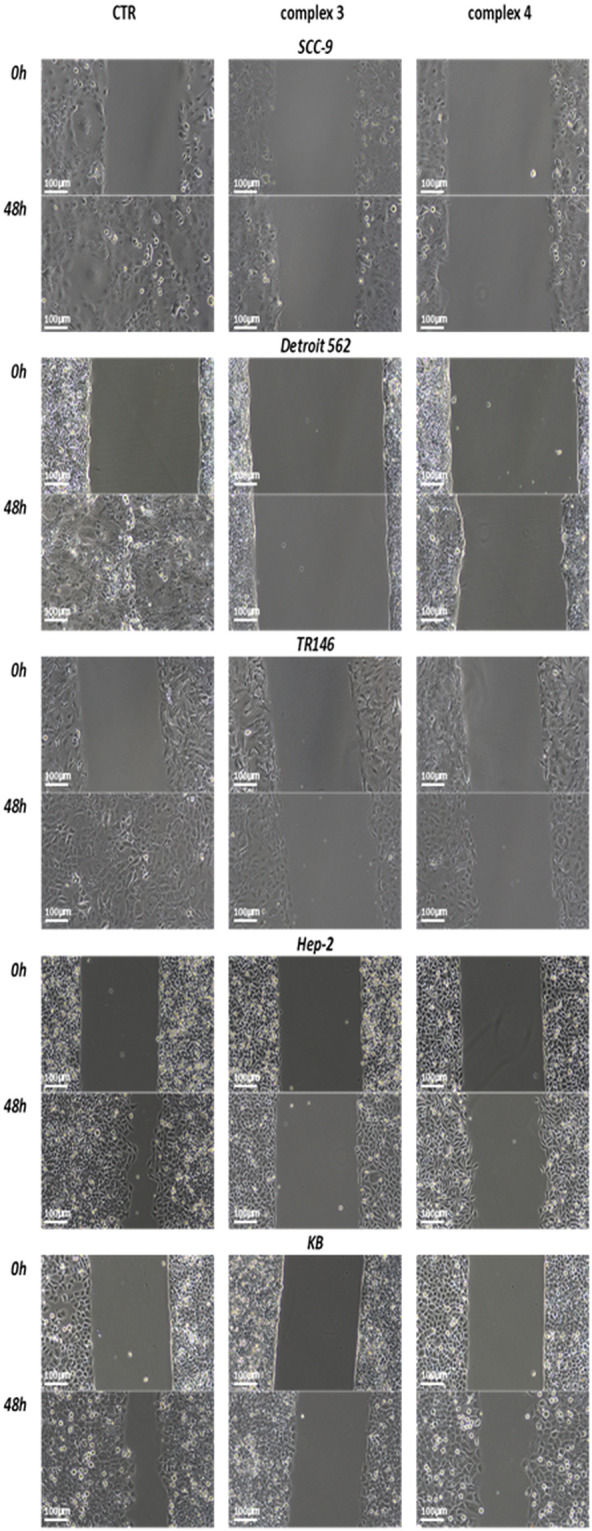
Microscopic imaging of cell migration and area closure at the beginning of the experiment (0 h) and after 48 h of the experiment. Imaging of treated cells (SCC-9, Detroit 562, TR146, Hep-2, and KB) with complex 3 or 4.

**Figure 7:**
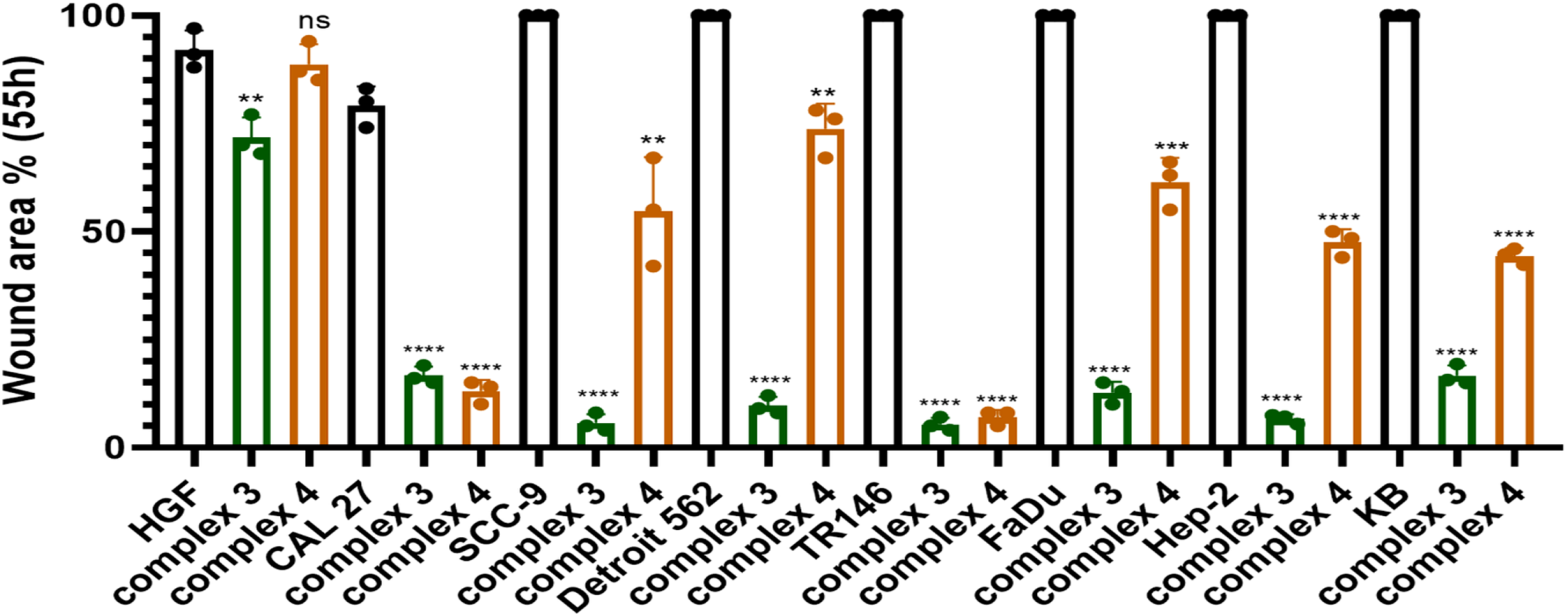
Graphical representation comparing the migration of cells treated with complex **3** or **4** (at IC_25_ concentration) to untreated controls after 55 h. Statistical analysis was performed using an unpaired *t*-test. Results are reported as ^**^p < 0.01, ^***^p < 0.001, and ^****^p < 0.0001.

To evaluate cell proliferation and migration, we employed *in vitro* assays, including the colony formation assay (**Figure 5**) and the wound healing assays (**Figures 6**, **7**). The colony formation assay assesses the ability of individual cells to grow into colonies, reflecting their long-term proliferative capacity. In contrast, the wound healing assay measures cell migration by quantifying the closure of a scratch introduced into a confluent cell monolayer. Together, these assays provide complementary insights into the proliferative and migratory behavior of cells under experimental conditions.

Similarly, like its cytotoxic effects, curcumin has also been studied for its impact on cell proliferation and migration, particularly in the context of HNC. In the CAL 27 cell line, curcumin reduced cell migratory capacity at concentrations as low as 50 µM and inhibited colony formation at concentrations starting from 12.5 µM (47, 55). In the SCC-9 cell line, curcumin markedly suppressed colony formation at 40 µM (49), while in the FaDu cell line, this effect was observed at 12.5 µM (55). In HPV-positive cell lines, curcumin also inhibited both colony formation and cell migration; for instance, in the Hep-2 cell line, significant inhibition was evident at 20 µM (56).

#### 3.4.1 Colony formation assay

CFA was used to evaluate the ability to form colonies after treatment with complex 3 or 4. The effect of both complexes on suppressing colony formation was comparable. However, complex 4 suppressed colony formation more effectively, as shown in **Table 2**. Complex 3 suppressed colony formation in SCC-9, Hep-2, and KB cell lines with a cloning efficiency of about 30%, indicating greater effectiveness in HPV+ cell lines. For complex 4, the highest suppression of colony formation was observed in the CAL 27 cell line, with an efficiency of 22%, and in Detroit 562 and Hep-2 cell lines, with an efficiency of around 30%. The graphical representation and comparison are shown in **Figure 5**, while the stained plates with formed colonies are presented in **Supplementary Figure S6**.

**Table 2:** Colony formation was analyzed using standard CFA.

Cloning efficiency (%)		
Cell line	Complex 3	Complex 4
*HGF*	95 ± 6	89 ± 2
*CAL 27*	39 ± 7	22 ± 3
*SCC-9*	34 ± 3	48 ± 4
*Detroit 562*	57 ± 8	25 ± 3
*TR146*	66 ± 19	42 ± 4
*FaDu*	73 ± 15	55 ± 10
*Hep-2*	33 ± 2	29 ± 1
*KB*	28 ± 2	94 ± 7

The cloning efficiency was determined after 24 h of treatment with complexes 3 and 4 at a concentration of IC_50_. Data are presented as the mean ± SD of four independent experiments (n = 4).

#### 3.4.2 Wound healing assay

An *in vitro* wound healing assay was performed to determine the effect of complex 3 or 4 on cell migration. The area of the wound bordered by a monolayer of cells was measured using ImageJ software. Wound closure was monitored from 0 to 55 h. **Table 3** shows the percentage of the area closed over time (a graphical representation can be seen in **Supplementary Figures S8**, **S9**). **Figure 6** shows microscope images at the beginning of the experiment (time 0 h) and after 48 h for selected cell lines (HPV-negative and HPV-positive), in which the strongest effect was observed. Microscope images after 24 h for all cell lines, as well as 55 h, can be seen in **Supplementary Figure S7**. **Figure 7** shows the graphical results of relative wound density from 0 to 55 h. The stained wound healing area after 55 h is shown in **Supplementary Figure S10**.

**Table 3:** Effect of complex 3 (C3) or complex 4 (C4) on cell migration over time (24 h, 48 h, 55 h).

Wound area (%)																								
*t* (h)	*HGF*			*CAL 27*			*SCC-9*			*Detroit 562*			TR146			FaDu			Hep-2			KB		
	*CTR*	*C3*	*C4*	*CTR*	*C3*	*C4*	*CTR*	*C3*	*C4*	*CTR*	*C3*	*C4*	*CTR*	*C3*	*C4*	*CTR*	*C3*	*C4*	*CTR*	*C3*	*C4*	*CTR*	*C3*	*C4*
24 h	27 ± 15	8 ± 3	13 ± 3	27 ± 1	10 ± 2	7 ± 4	62 ± 4	5 ± 2	17 ± 12	51 ± 7	8 ± 2	13 ± 7	55 ± 3	3 ± 1	3 ± 1	44 ± 1	7 ± 1	24 ± 6	56 ± 7	3 ± 1	9 ± 2	61 ± 7	7 ± 1	16 ± 4
48 h	62 ± 5	13 ± 5	47 ± 11	53 ± 2	15 ± 3	11 ± 3	100 ± 0	6 ± 2	37 ± 8	100 ± 0	9 ± 3	38 ± 11	100 ± 0	4 ± 1	5 ± 1	80 ± 4	9 ± 1	43 ± 10	93 ± 1	5 ± 1	23 ± 3	92 ± 1	13 ± 1	33 ± 4
55 h	95 ± 3	71 ± 2	91 ± 1	75 ± 4	16 ± 3	12 ± 3	100 ± 0	7 ± 2	55 ± 13	100 ± 0	10 ± 2	76 ± 2	100 ± 0	5 ± 1	7 ± 1	100 ± 0	12 ± 1	61 ± 6	100 ± 0	7 ± 1	47 ± 2	100 ± 0	17 ± 2	44 ± 2

Wound area closure was monitored after treatment with complex 3 or 4, compared to control (nontreated cells). Data are presented as the mean ± SD of three independent experiments (*n* = 3).

Although complex 4 demonstrated a stronger effect in suppressing colony formation, complex 3 showed superior inhibition of cell migration. After 55 h, area closure was less than 10% in SCC-9, TR146, and Hep-2 cell lines, and less than 20% in CAL 27, Detroit 562, FaDu, and KB cell lines. Thus, complex 3 exhibited a very strong ability to suppress migration in both HPV− and HPV+ HNSCC cell lines.

The effect of native curcumin on cell migration has been investigated across multiple head and neck cancer cell models. Ma et al. demonstrated that curcumin at 100 μM significantly inhibits migration in CAL 27 cells (47), and Ohnishi et al. reported a comparable outcome in OSCC cells treated with 15 μM curcumin (57). Similarly, Ardito et al. confirmed that curcumin suppresses migration in tongue squamous cell carcinoma (TSCC) cells (58). Together, these studies indicate that curcumin has a reproducible inhibitory effect on cell migration in head and neck cancer cell lines.

In our study, complex 4 exhibited significant migrastatic effects in CAL 27 and TR146 cells, with less than 15% wound closure after 55 h (**Figure 7**). A significant reduction in migration was also observed in the HPV+ Hep-2 and KB cell lines, both showing less than 50% closure at the same time point. Complex 3 showed the strongest overall effect, reducing wound closure to below 10% in SCC-9, TR146, and Hep-2 cells after 55 h.

### 3.5 Immunomodulatory activity of complexes 3 and 4

**Figure 8:**
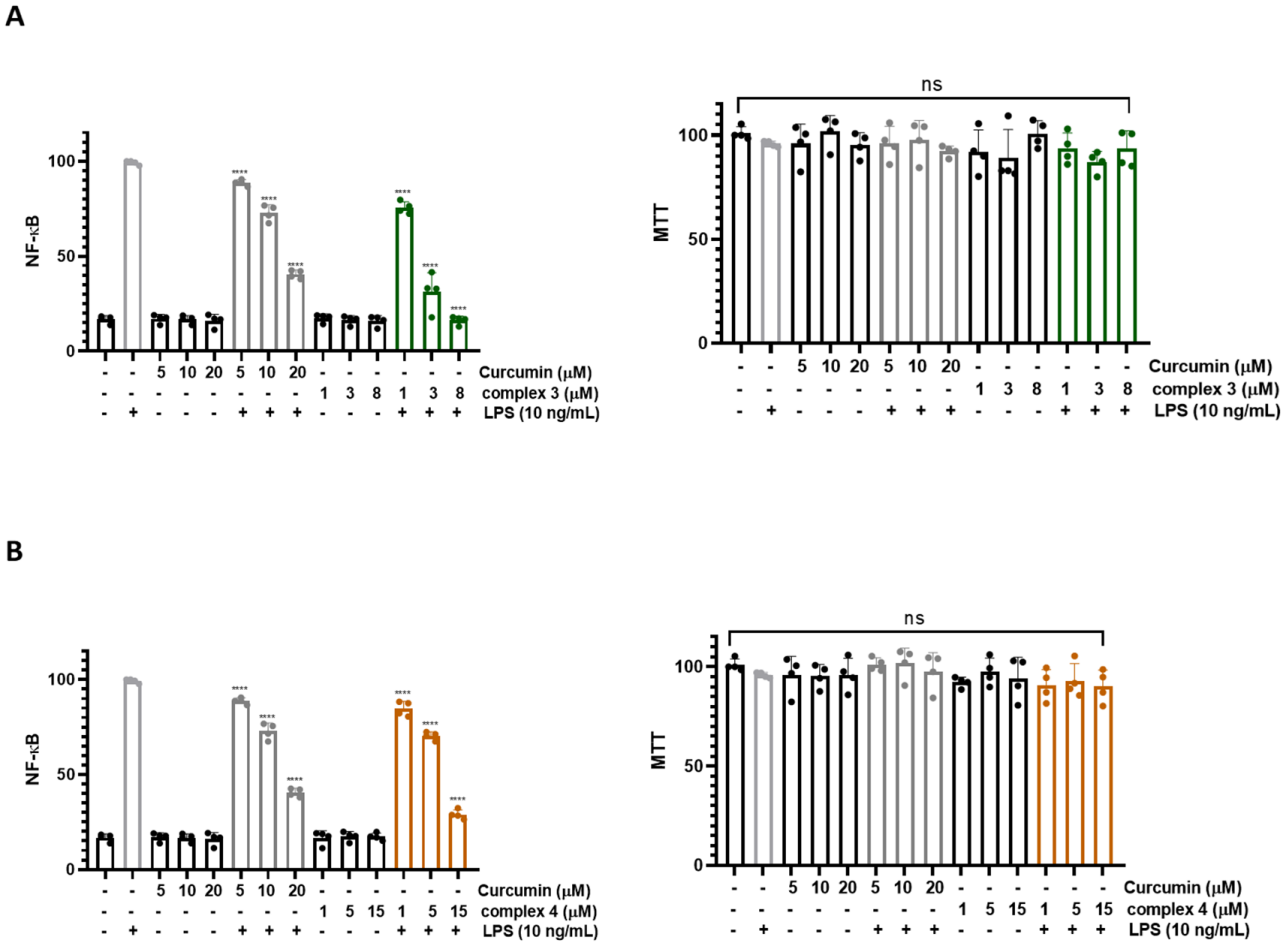
**(A)** Inhibition of NF-κB activation using complex 3. (Left) THP1-Blue NF-κB cells were treated with complex 3 (1–15 μM) and curcumin (5–20 μM). In both cases, there is a significant reduction in the activation of NF-κB. The MTT viability assay (right) was used to analyze the toxic effect of complex 3 on THP1-Blue NF-κB cells. **(B)** Inhibition of NF-κB activation using complex 4. (Left) THP1-Blue NF-κB cells were treated with complex 4 (1–15 μM) and curcumin (5–20 μM). In both cases, there is a significant reduction in the activation of NF-κB. The MTT viability assay (right) was used to analyze the toxic effect of complex 4 on THP1-Blue NF-κB cells. Data are presented as the mean ± SEM of four independent experiments (n = 4). **(B)** Statistical analysis was performed using a one-way ANOVA with Dunnett’s multiple comparison tests. Results are reported as not significant (ns) and ^****^p < 0.0001.

We investigated the effect of complexes 3 and 4 on LPS-induced NF-κB activation in THP1-Blue NF-κB cells (**Figure 8**). Complex 3 showed the most significant inhibition at 8 μM, reducing NF-κB activation by approximately 80% (**Figure 8A**). Complex 4 suppressed NF-κB activation by about 70% at a concentration of 15 μM (**Figure 8B**). Native curcumin was also tested for comparison, showing approximately 60% suppression at 20 μM. Complex 3 at 8 µM suppressed NF-κB activation roughly four times more effectively than native curcumin at 10 µM. Even at 3 µM, complex 3 inhibited NF-κB activation about twice as effectively as curcumin at 10 µM. Complex 4 at 5 µM produced a similar suppression as curcumin at twice the concentration (10 µM). At 15 µM, however, complex 4 inhibited NF-κB activation approximately twice as effectively as curcumin at 20 µM.

Neither complex 3, complex 4, nor curcumin exhibited significant cytotoxicity in THP1-Blue NF-κB cells; therefore, the observed inhibition of NF-κB activation is unlikely to result from cytotoxic effects.

**Figure 9:**
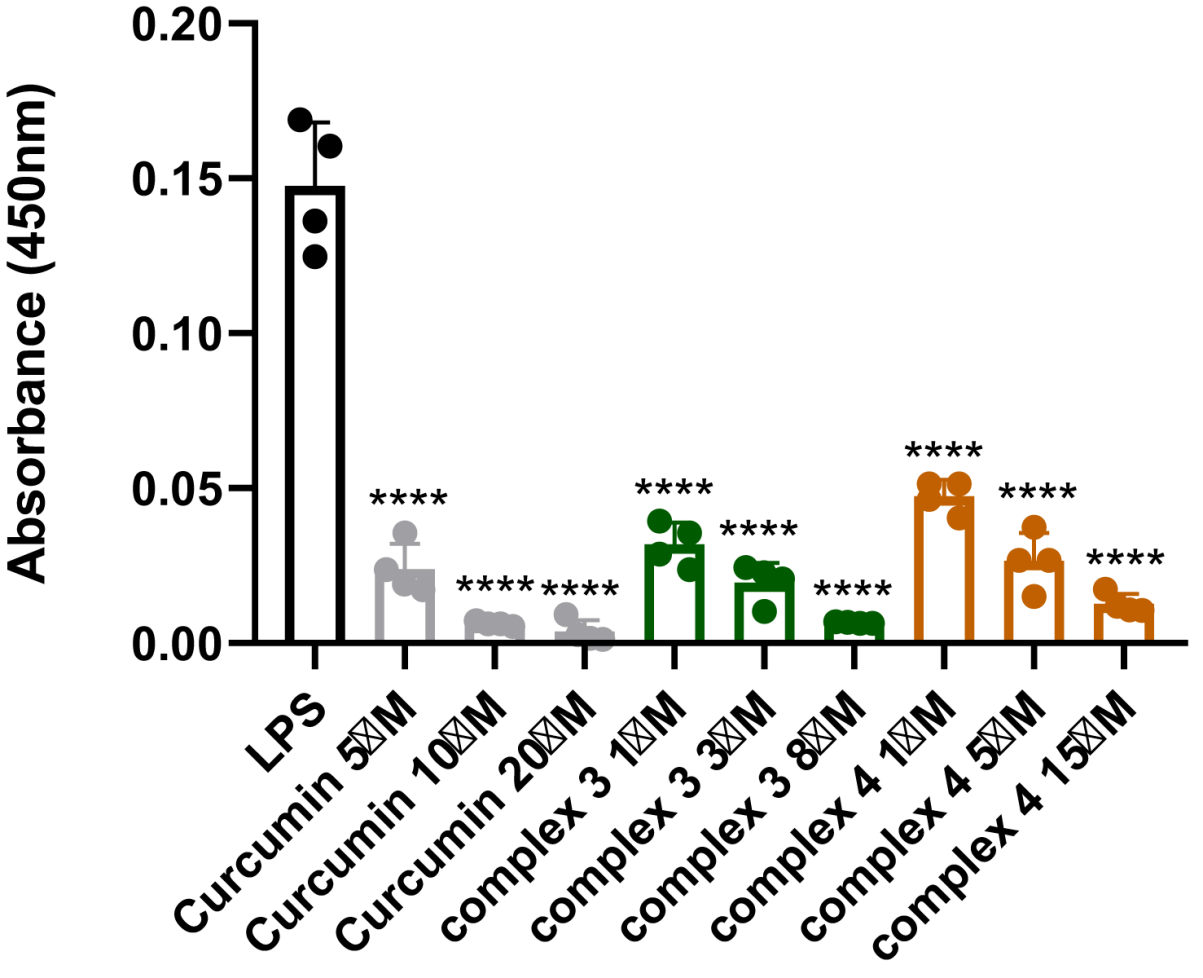
Detection of NF-κB (p65) in nuclear extracts after treatment with complex 3 (5–20 μM), complex 4 (1–15 μM), and curcumin (5–20 μM). Data are presented as the mean ± SEM of four independent experiments (n = 4). Statistical analysis was performed using a one-way ANOVA with Dunnett’s multiple comparison tests. Results are reported as ^****^p < 0.0001.

The effects of complexes 3 and 4 on NF-κB (p65) DNA-binding activity were assessed in cellular extracts (**Figure 9**). Both complexes showed significant inhibition at 1 μM, reducing NF-κB (p65) levels by approximately 70%, whereas curcumin exhibited a similar effect only at 5 μM. Complex 3 displayed the strongest activity, with less than 5% NF-κB (p65) detected in cellular extracts at 8 μM. These findings indicate that both complexes strongly interfere with NF-κB (p65) nuclear translocation or DNA binding in a dose-dependent manner, underscoring their potential as effective modulators of NF-κB-driven transcription. Curcumin showed comparable effects but only at higher concentrations.

**Figure 10:**
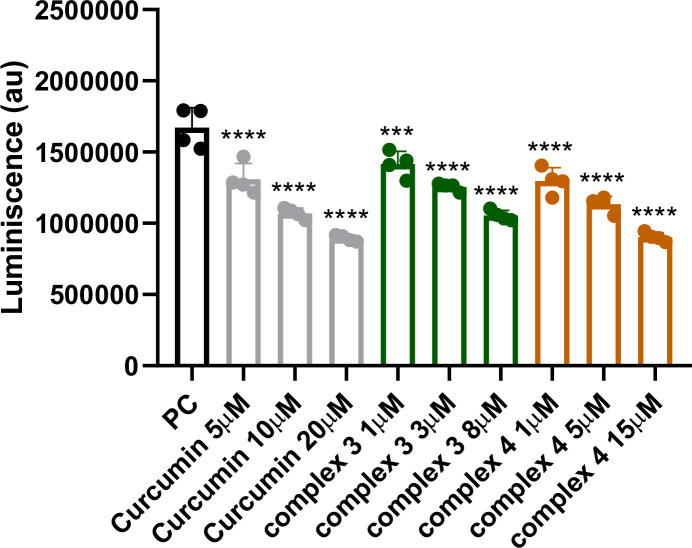
Detection of IKKβ inhibition after treatment with complex 3 (5–20 μM), complex 4 (1–15 μM), and curcumin (5–20 μM). Data are presented as the mean ± SEM of four independent experiments (*n* = 4). Statistical analysis was performed using a one-way ANOVA with Dunnett’s multiple comparison tests. Results are reported as ^***^p < 0.001 and ^****^p < 0.0001.

IKKβ is a key protein responsible for activating the NF-κB signaling pathway. To determine whether complexes 3 and 4 affect this pathway, we measured IKKβ kinase activity (**Figure 10**). As expected, both complexes, as well as native curcumin, reduced IKKβ activity in a dose-dependent manner. Complex 4 and curcumin showed nearly comparable effects, but at different concentration ranges. At 1 μM, complex 4 inhibited IKKβ activity by approximately 30%, a level of inhibition that curcumin achieved only at a fivefold higher concentration. At 8 μM, complex 3 reduced IKKβ activity by about 40%. These results suggest that both complexes 3 and 4 can effectively interfere with NF-κB signaling at the level of IKKβ, contributing to their overall anti-inflammatory and potential anticancer effects.

**Figure 11:**
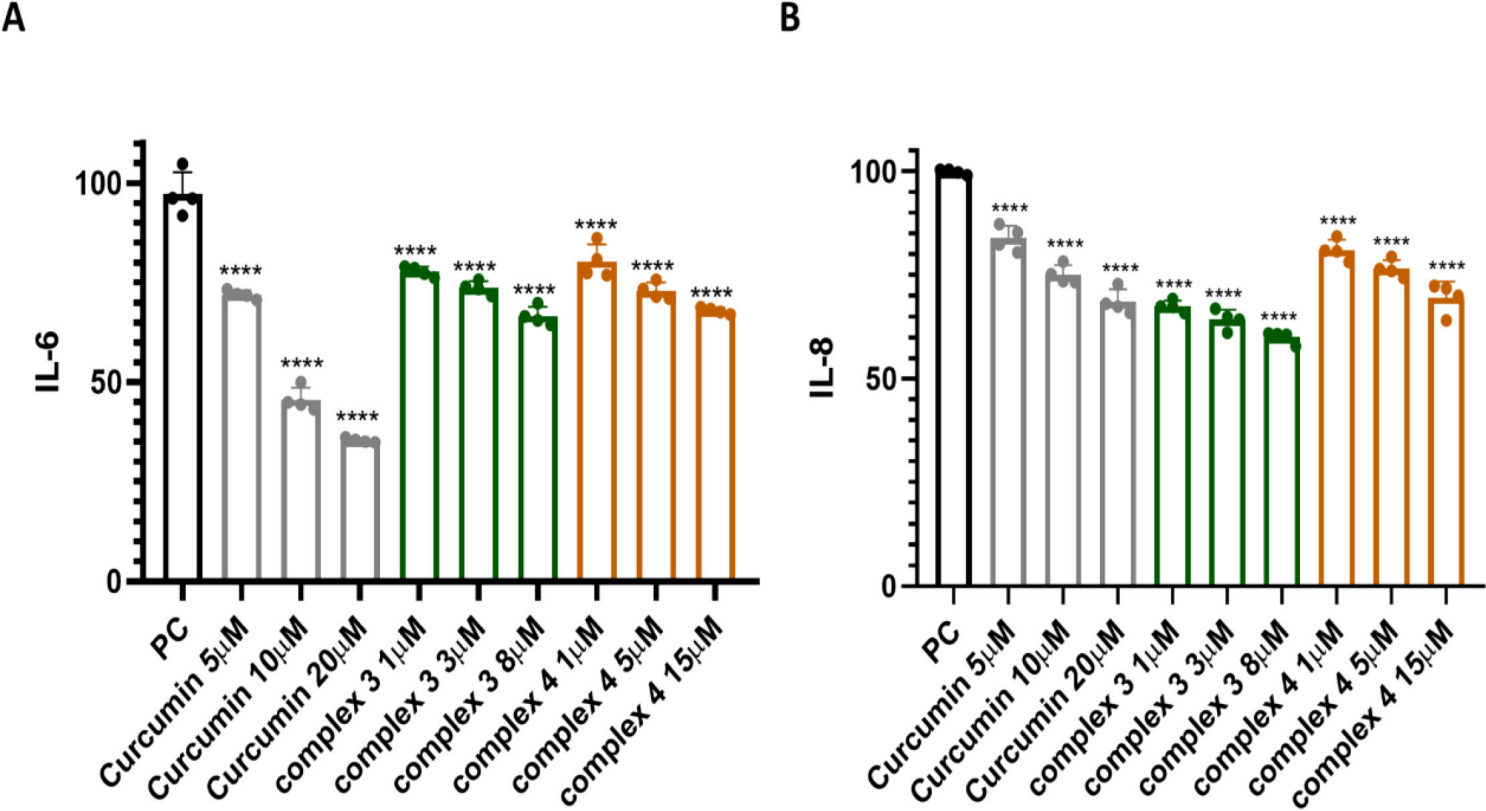
A sandwich ELISA kit was used to determine the effect of complexes 3 and 4 on **(A)** IL-6 and **(B)** IL-8. Native curcumin was used for comparison. Data are presented as the mean ± SEM of four independent experiments (n = 4). **(B)** Statistical analysis was performed using a one-way ANOVA with Dunnett’s multiple comparison tests. Results are reported as not significant (ns) and ^****^p < 0.0001.

We also investigated the effect of complexes 3 and 4 on IL-6 using an IL-6 ELISA kit as a sandwich assay (see **Figure 11A**). The effect on IL-6 was observed for both complexes 3 and 4, as well as for native curcumin. At a concentration of 8 μM, complex 3 decreased IL-6 concentration by approximately 35%. A similar effect on IL-6 was observed for complex 4, which decreased IL-6 by approximately 30% at a concentration of 15 μM. An effect on IL-6R was also investigated (**Supplementary Figure S10**), but the decrease in IL-6R concentration was only about 15% for both complexes 3 and 4 at the highest concentrations (8 and 15 μM, respectively).

Furthermore, the effect on IL-8 was investigated using a sandwich ELISA kit (**Figure 11B**). Complex 3 showed a stronger effect on IL-8 than curcumin alone. A reduction in IL-8 concentration was observed already at 1 µM of complex 3. At 8 µM, complex 3 decreased IL-8 concentration by approximately 40%. Complex 4 showed a similar effect to native curcumin, with a 15-µM concentration reducing IL-8 by approximately 30%. Complex 3 reduced IL-8 levels at 1 µM roughly 1.5-fold more effectively than curcumin at a fivefold higher concentration (5 µM). Moreover, even 20 µM native curcumin did not decrease IL-8 levels as effectively as 1 µM of complex 3. Complex 4 showed a similar effect on IL-8 levels at 5 µM as curcumin at twice the concentration (10 µM).

The obtained results showed that the tested complexes 3 and 4, along with curcumin, significantly decreased the protein levels of IL-6d and IL-8d in the media, as detected by an ELISA kit. This reduction is likely due to the direct interaction of these compounds with their protein partners, confirming the predicted direct interactions of complexes 3 and 4, as well as curcumin, with IL-6d and IL-8d, as suggested by *in silico* docking studies. Additionally, these complexes exhibited a potent inhibitory effect on NF-κB activation, which is associated with the inhibition of IKKβ and the nuclear translocation of p65. This suggests that the anticancer effects of complexes 3 and 4 may be linked to their multitargeting capabilities, specifically toward the IL-6d, IL-8d, and NF-κB signaling pathways.

## 4 Discussion

In this study, two Ru(II) derivatives of curcuminoids were synthesized. The structures of the synthesized complexes (complexes 3 and 4) were confirmed by NMR spectroscopy. The purity of the resulting complexes was confirmed by NMR and HPLC/MS methods. Additionally, UV–Vis spectroscopy confirmed that, under the measurement conditions in a PBS environment, no significant aggregation, dissociation, or other interactions occurred.

We investigated the anticancer effect of complexes 3 and 4 on selected HNC cell lines. Complex 3 exhibited higher cytotoxic compared to complex 4. Complex 3 was cytotoxic against both HPV− and HPV+ lines, whereas complex 4 demonstrated selectivity toward the SCC-9 line. Complex 3 showed cytotoxic effects within a concentration range of 10–20 μM. In contrast, complex 4 exhibited cytotoxicity only at concentrations above 20 μM, except in the SCC-9 line, where the IC_50_ was 14.30 μM.

Studies suggest that curcumin derivatives exhibit better cytotoxic effects than native curcumin. Specifically, in HNC, studies on the Hep-2 cell line found that curcuminoids have an IC_50_ approximately twofold lower than that of curcumin alone (28, 56).

Also, ruthenium derivatives exhibit higher cytotoxic effect than native curcumin, and even higher than cisplatin, as shown in the study by Li et al., where their two complexes had IC_50_ values of 3.8 and 2.1 μM, compared to curcumin and cisplatin, which had IC_50_ values of 13.1 and 13.8 μM, respectively (A549 cell line used) (33).

In terms of cell proliferation and migration, complex 3 shows similar results to complex 4. Complex 3 exhibits the strongest suppression of proliferation in HPV+ cell lines (Hep-2 and KB) and in OSCC lines, where proliferation is reduced by more than 60%. Complex 4, on the other hand, most effectively inhibits proliferation in the CAL 27 cell line, with a cloning efficiency of only 22%. In the SCC-9 cell line, where complex 4 demonstrates the highest cytotoxicity, proliferation is also significantly suppressed, with a posttreatment cloning efficiency of 48%.

However, complex 3 inhibited cell migration more effectively than complex 4. Complex 3 suppressed migration across all cell lines, with area closure remaining below 20% even after 55 h. Complex 4 demonstrated its strongest effect on the CAL 27 cell line, where area closure was less than 15%. Similar inhibitory effects of complex 4 were also observed in the TR146 cell line, a human neck metastasis model.

Curcumin and its derivatives are known to suppress cell proliferation and migration in HNSCC. Metastatic activity in HNSCC is significantly associated with NF-κB activation (56, 59). Activation and upregulation of NF-κB in HNSCC are often caused by chronic exposure to carcinogens from cigarette smoke or the progression of HPV-infected cells (60). Activation of NF-κB downregulates genes involved in cell proliferation, chemotherapeutic resistance, proinflammatory responses, and metastasis, including IL-6 and IL-8. Inhibition of NF-κB in HNSCC leads to reduced tumor growth and decreased expression of IL-6 and IL-8 (60, 61). Cohen et al. studied the suppression of IL-6 and IL-8 in HNSCC following curcumin treatment and found that curcumin reduced IL-6 and IL-8 levels in a concentration-dependent manner (62). Similarly, the curcumin derivative BDMC-A reduced NF-κB, IL-6, and IL-8 levels in the Hep-2 line and effectively inhibited markers of invasion, angiogenesis, and metastasis than native curcumin (28).

*In silico* studies have shown that both complexes 3 and 4 have strong affinity for NF-κB, IL-8, and IL-6, while complex 4 showed the strongest affinity for IL-6 and NF-κB (− 11.69 and − 10.56 kcal/mol, respectively). For native curcumin, compound 1, and compound 2, which do not contain Ru(II) in their structure, the affinity for all three ligands studied is lower than for complexes 3 and 4, which do contain Ru(II). The effect of complexes 3 and 4 on NF-κB, IL-6, and IL-8 *in vitro* was observed in comparison with native curcumin. Both complexes inhibited NF-κB activation better than native curcumin. Complex 3 inhibited NF-κB activation at a concentration of 8 μM by about 80%, and complex 4 inhibited activation by about 70% at a concentration of 15 μM. Native curcumin inhibited NF-κB activation by about 60% at concentrations up to 20 μM. The effects of complexes 3 and 4 on NF-κB (p65) DNA-binding activity in nuclear extracts were also evaluated. Both complexes strongly interfered with NF-κB (p65) nuclear translocation even at 1 µM, reducing its translocation into the nucleus by approximately 70% and thereby suppressing NF-κB (p65) activation. To further explore the mechanism, the inhibitory effect on IKKβ—a key protein that activates the NF-κB signaling pathway—was also assessed. As expected, both complexes inhibited IKKβ kinase activity. Complex 4 suppressed IKKβ activation by approximately 30% at 1 µM, while complex 3 inhibited IKKβ activity by around 40% at 8 µM. These findings indicate that both complexes 3 and 4 can effectively interfere with NF-κB signaling at the level of IKKβ. However, a limitation of this approach is that the inhibitory effects were studied *in vitro* using monocytes, which may not fully represent *in vivo* conditions. A similar approach was employed in previous studies (63–66). Future experiments will also utilize HNSCC models. Monocytes, in particular, play a significant role in HNSCC pathogenesis. A meta-analysis by Wei et al. demonstrated that HNSCC patients with a higher lymphocyte-to-monocyte ratio exhibit significantly better therapeutic outcomes (67). Similarly, Heimdal et al. reported that increased IL-6 secretion from monocytes of HNSCC patients (following endotoxin stimulation) was associated with lower overall disease-free survival (68).

The effects of complexes 3 and 4 on IL-6 were also investigated. Both complexes produced a statistically significant reduction in IL-6 concentration of approximately 30%, using 8 μM for complex 3 and 15 μM for complex 4. Regarding IL-8, complex 3 exhibited the greatest reduction, decreasing IL-8 by approximately 40% at 8 μM. Complex 4 (15 μM) showed a similar effect to native curcumin (20 μM), reducing IL-8 concentration by around 30%. Further experiments are required to confirm the direct inhibitory effects on IL-6 and IL-8, which will be addressed in subsequent follow-up studies.

Given the studied anti-inflammatory and antitumor activities of complexes 3 and 4 in HNC models, future studies should focus on further elucidating their precise mechanisms of action. In particular, comprehensive pathway analyses, such as transcriptomic and proteomic profiling, are needed to confirm the extent of NF-κB pathway modulation and to uncover additional downstream mediators involved in tumor suppression. Direct inhibitory effects on IL-6 and IL-8 will also need to be investigated in dedicated studies. For example, repression of IL-6/STAT3 signaling axis is associated with overexpression of wild-type p53 (69). In the case of Ru(II) curcumin, it has been reported that its effect (in various cancer models) is associated with activation of wild-type p53 and Nuclear Factor Erythroid 2–Related Factor 2 (NRF2) signaling, as well as a reduction in the protein level of mutated oncogenic p53. Nevertheless, in this case, ROS levels were decreased, most probably due to NRF2 overexpression (18).

Furthermore, structure–activity relationship (SAR) studies will be crucial to identify the key pharmacophoric features responsible for the observed selectivity of complex 4 toward OSCC and the broader efficacy of complex 3 across both HPV+ and HPV− HNC cell lines. This knowledge will inform the rational design of next-generation analogs with optimized binding characteristics, enhanced cellular uptake, and improved pharmacokinetic profiles. Future work will also explore combination therapies of these complexes with existing chemotherapeutics, along with *in vivo* experiments in mouse models.

Overall, these findings support the advancement of complexes 3 and 4 as promising candidates for targeting inflammation-driven tumor progression in head and neck cancers.

## 5 Conclusion

Newly synthesized ruthenium-enhanced curcumin derivatives, complexes 3 and 4, demonstrated enhanced cytotoxicity, antiproliferative activity, and antimigratory effects, all of which were observed against selected HNC cell lines, including both HPV-positive and HPV-negative subtypes. Complex 3 showed broader activity, including efficacy against HPV+ lines, while complex 4 exhibited selectivity toward OSCC. Both complexes effectively suppressed the key proinflammatory and protumorigenic NF-κB signaling pathway, and the observed effects on IL-6 and IL-8 levels suggest a potential mechanistic link between their anticancer and anti-inflammatory properties, although direct inhibitory effects on IL-6 and IL-8 have not yet been confirmed. These findings indicate that ruthenium(II)–curcuminoid complexes represent promising structural scaffolds for the development of novel therapeutic strategies targeting tumor growth, inflammation, and metastatic potential in HNC.

## Data Availability

The raw data supporting the conclusions of this article will be made available by the authors, without undue reservation.
