# Supplementary material for: Ruthenium-enhanced curcumin derivatives target tumor growth and cancer-related inflammation in head and neck cancer models
Source: Front Oncol. 2025 Dec 17;15:1708944. doi: 10.3389/fonc.2025.1708944 (PMC12754617; doi:10.3389/fonc.2025.1708944)
Supplement: Supplementary file 1 [file DataSheet1.docx]

**Ruthenium-Enhanced Curcumin Derivatives Targets Tumor Growth and Cancer-Related Inflammation in Head and Neck Cancer Models**

***Kateřina Veselá^1,2^, Ameneh Tatar^1,2^, Zdeněk Kejík^1,2^, Nikita Abramenko^1^, Robert Kaplánek^1^, Petr Babula^3,4^, Kateřina Kučnirová^1^, Jan Hajduch^1^, Pavel Martásek^1,2^ and Milan Jakubek^1,2*^***

*^1^BIOCEV, Biotechnology and Biomedicine Center of the Academy of Sciences and Charles University in Vestec, Vestec, Czechia*

*^2^Department of Paediatrics and Inherited Metabolic Disorders, First Faculty of Medicine, Charles University and General University Hospital in Prague, Prague, Czechia*

*^3^Department of Pathological Physiology, Faculty of Medicine, Masaryk University, Kamenice 5, 625 00 Brno, Czech Republic*

*^4^Department of Physiology, Faculty of Medicine, Masaryk University, Kamenice 5, 625 00 Brno, Czech Republic*

***Correspondence:**

Milan Jakubek; [Milan.Jakubek@lf1.cuni.cz](mailto:Milan.Jakubek@lf1.cuni.cz)

**Keywords: Ru-complex, curcumin, anti-inflammatory, anticancer, head and neck carcinoma**

# **Cell lines information**

***HGF***, Human Gingiva Fibroblast, (ATCC, USA) were cultured in DMEM:F12 (Gibco, Life Technology Itd., Renfrew, UK) and the media was supplemented with 5% (v/v) FBS, qualified, US (Gibco, Life Technology Itd., Renfrew, UK) and 1% (v/v) penicillin - streptomycin antibiotics (Gibco, Life Technology Itd., Renfrew, UK) at 37 °C in 5% CO_2_.

***CAL 27***, Tongue Squamous Cell Carcinoma, (ATCC, USA) were cultured in DMEM (Gibco, Life Technology Itd., Renfrew, UK) and the media was supplemented with 10% (v/v) FBS, qualified, US (Gibco, Life Technology Itd., Renfrew, UK) and 1% (v/v) penicillin - streptomycin antibiotics (Gibco, Life Technology Itd., Renfrew, UK) at 37 °C in 5% CO_2_.

***SCC-9***, Tongue Squamous Cell Carcinoma, (ATCC, USA) were cultured in DMEM:F12 (Gibco, Life Technology Itd., Renfrew, UK) and the media was supplemented with 10% (v/v) FBS, qualified, US (Gibco, Life Technology Itd., Renfrew, UK), 1% (v/v) penicillin - streptomycin antibiotics (Gibco, Life Technology Itd., Renfrew, UK) and hydrocortisone (Sigma-Aldrich, Merck, Germany) at 37 °C in 5% CO_2_.

***Detroit 562***, Pharynx carcinoma, (ATCC, USA) were cultured in RPMI 1640 (Gibco, Life Technology Itd., Renfrew, UK) and the media was supplemented with 10% (v/v) FBS, qualified, US (Gibco, Life Technology Itd., Renfrew, UK) and 1% (v/v) penicillin - streptomycin antibiotics (Gibco, Life Technology Itd., Renfrew, UK) at 37 °C in 5% CO_2_.

***FaDu***, Pharynx Squamous Cell Carcinoma, (ATCC, USA) were cultured in DMEM:F12 (Gibco, Life Technology Itd., Renfrew, UK) and the media was supplemented with 10% (v/v) FBS, qualified, US (Gibco, Life Technology Itd., Renfrew, UK) and 1% (v/v) penicillin - streptomycin antibiotics (Gibco, Life Technology Itd., Renfrew, UK) at 37 °C in 5% CO_2_.

***TR146***, Bucal Carcinoma, (Sigma-Aldrich, Merck, Germany) were cultured in Ham´s F12 (Gibco, Life Technology Itd., Renfrew, UK) and the media was supplemented with 10% (v/v) FBS, qualified, US (Gibco, Life Technology Itd., Renfrew, UK) and 1% (v/v) penicillin - streptomycin antibiotics (Gibco, Life Technology Itd., Renfrew, UK) at 37 °C in 5% CO_2_.

***Hep-2***, Epidermoid Carcinoma of the Larynx (HeLa), (ATCC, USA) were cultured in EMEM (Gibco, Life Technology Itd., Renfrew, UK) and the media was supplemented with 10% (v/v) FBS, qualified, US (Gibco, Life Technology Itd., Renfrew, UK) and 1% (v/v) penicillin - streptomycin antibiotics (Gibco, Life Technology Itd., Renfrew, UK) at 37 °C in 5% CO_2_.

***KB***, Epidermal Carcinoma of the Mouth (HeLa), (Cytion, Germany) were cultured in EMEM (Gibco, Life Technology Itd., Renfrew, UK) and the media was supplemented with 10% (v/v) FBS, qualified, US (Gibco, Life Technology Itd., Renfrew, UK) and 1% (v/v) penicillin - streptomycin antibiotics (Gibco, Life Technology Itd., Renfrew, UK) at 37 °C in 5% CO_2_.

***THP1-Blue NF-κB Cells***, NF-κB SEAP Reporter Monnocytes (InvivoGen, USA) were cultured in RPMI 1640 (Gibco, Life Technology Itd., Renfrew, UK) and the media was supplemented with 10% (v/v) heat-inactivated FBS (Gibco, Life Technology Itd., Renfrew, UK) and 100 μg/mL Normocin and 1% (v/v) penicillin - streptomycin antibiotics (Gibco, Life Technology Itd., Renfrew, UK) at 37 °C in 5% CO_2_.

**Docking studies**


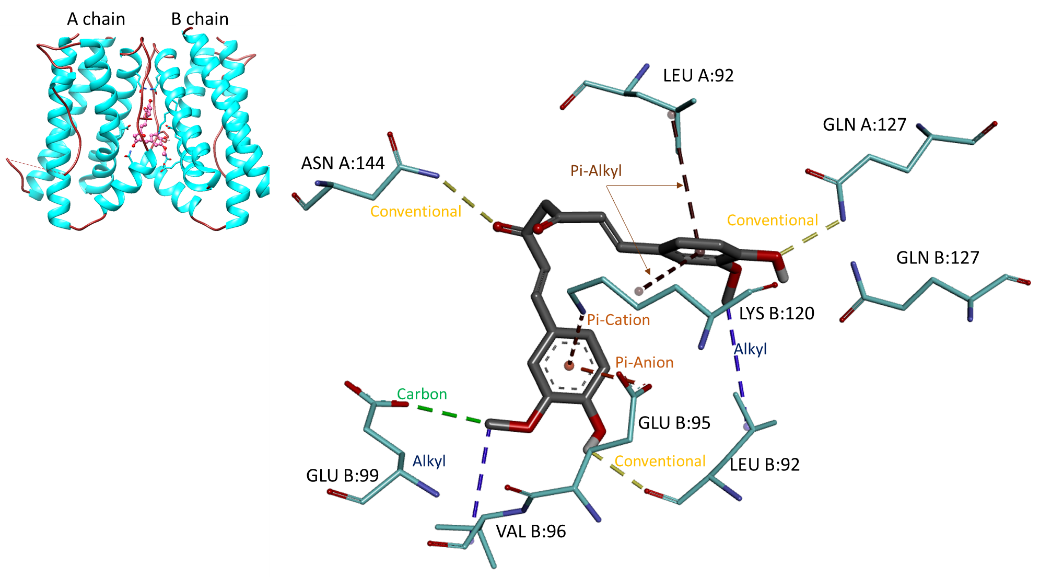


*Figure S1 Docking poses of native curcumin (-8.62 kcal/mol) in the dimeric form of IL-6. Upper left inset provides a global view of the IL-6 dimer in cartoon representation.*


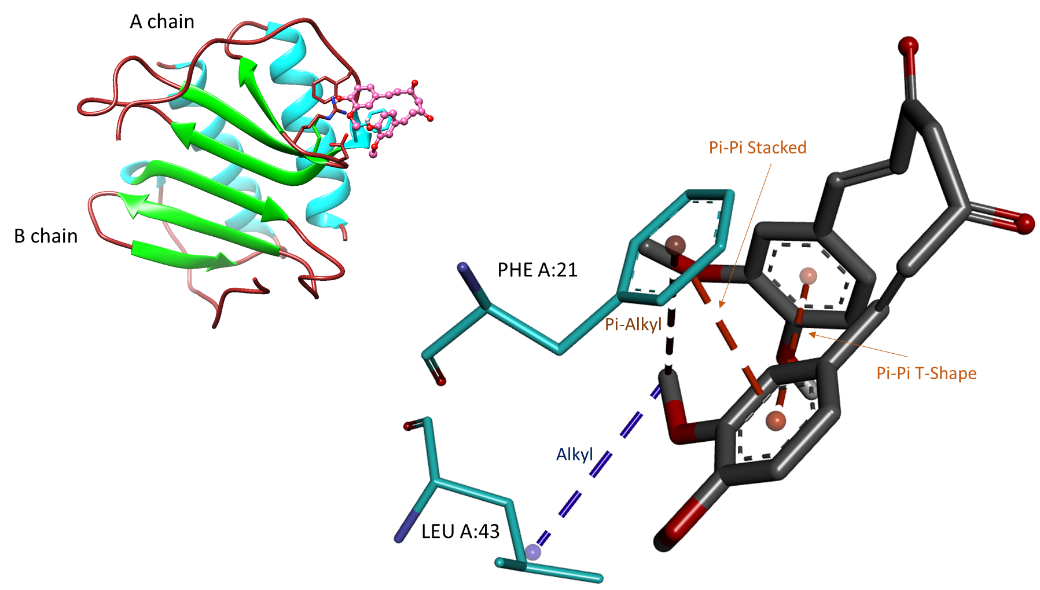


*Figure S2 Docking poses of native curcumin (-5.44 kcal/mol) in the dimeric form of IL-8. Upper left inset provides a global view of the IL-8 dimer in cartoon representation.*


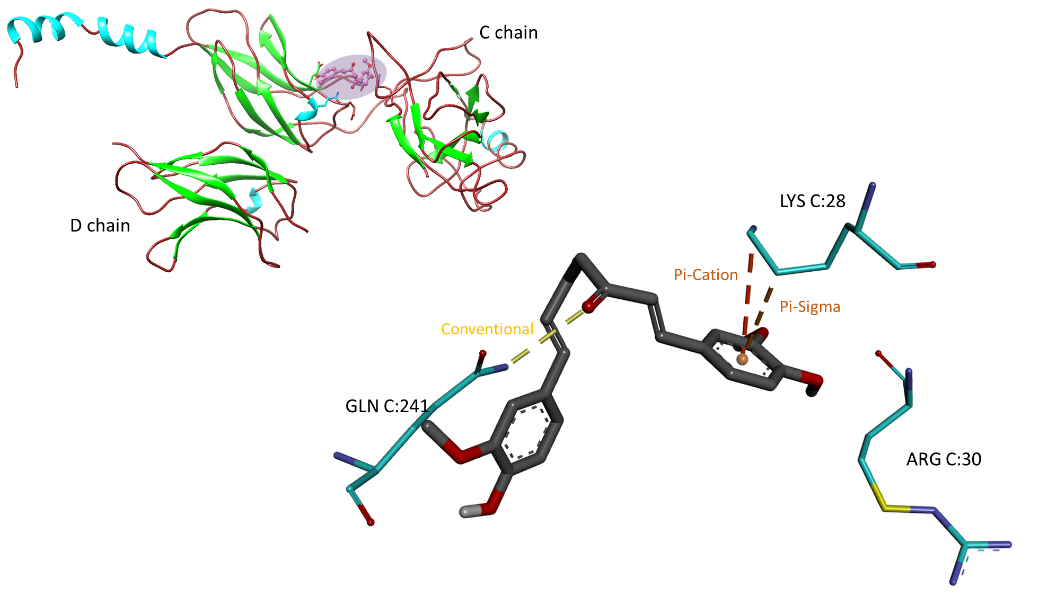


*Figure S3 Docking poses of native curcumin (-5.36 kcal/mol) in the NF-κB. Upper left inset provides a global view of NF-κB in cartoon representation.*

Table S1 Results of predicted binding energy of complex **3** and complex **4** ligands with IL-6R. Including comparison of the probable binding energy of complex **3** and complex **4** (ligand **1** and ligand **2**) without Ru in the structure.

| **Receptor** | **Binding Energy (kcal /mol)** | | | |
| --- | --- | --- | --- | --- |
|  | **Complex 3** | **Compound 1** | **Complex 4** | **Compound 2** |
| IL-6R | -12.08 | -9.29 | -7.68 | -6.90 |

**A**


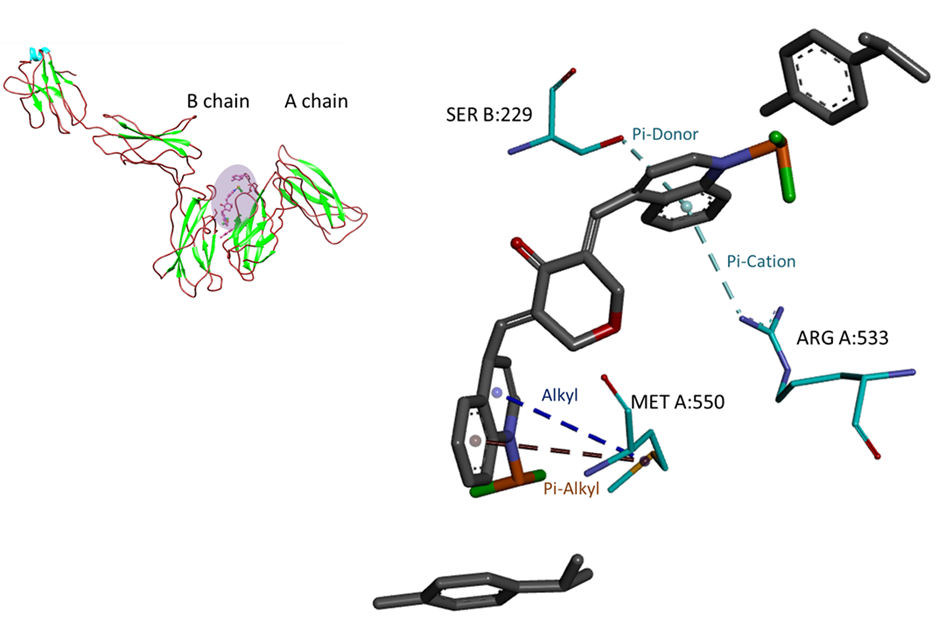


**B**


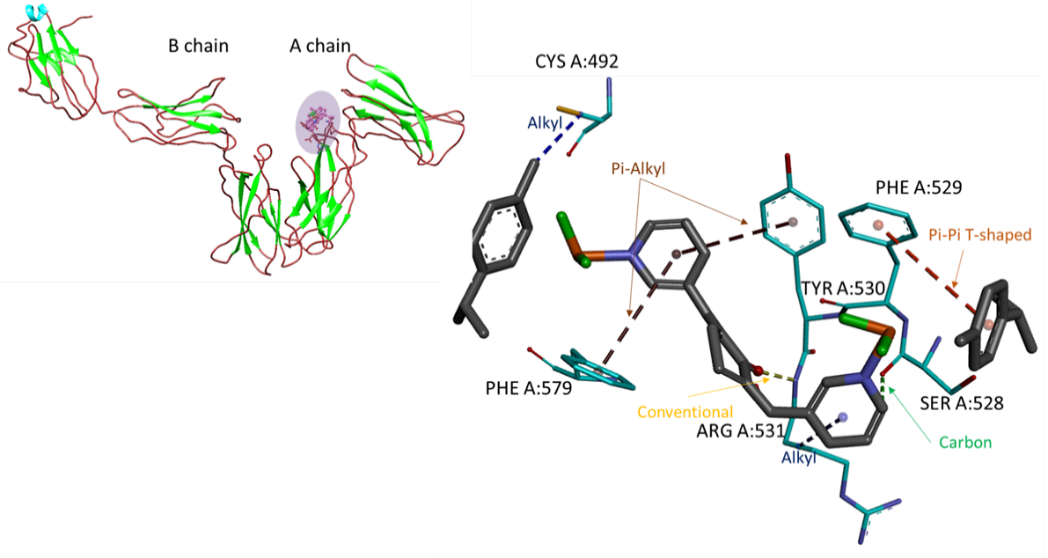


Figure S4 Docking pose of **A)** complex **3** ( -12.08 kcal/mol) **B)** complex **4** (-7.68 kcal/mol) on the surface of the IL-6R (colored magenta in violet area). Upper left inset provides a global view of the IL-6R in cartoon representation (cyan helices, brown coils and green strands). The zoomed panel on the right details the specific non-covalent contacts stabilizing this pose with interaction types color-coded as follows: brown dashed lines - π-alkyl, blue dashed lines – alkyl, yellow dashed lines – conventional hydrogen bond, green dashed lines – carbon hydrogen bond, orange dashed line - π- π T-shaped contact, light blue dashed lines – π-cation and π-donor bonds.

**MTT viability assay**

Table S2 MTT viable count assay was used to determine the cytotoxic effect. The IC_50_ values of complex **3** and complex **4** after 48h and 72h treatment was determined and the selectivity index after 24h of treatment was evaluated.

| **complex 3** | | | | **complex 4** | | |
| --- | --- | --- | --- | --- | --- | --- |
|  | ***IC_50_ 48h*** | ***IC_50_ 72h*** | ***Selectivity index 24h*** | ***IC_50_ 48h*** | ***IC_50_ 72h*** | ***Selectivity index 24h*** |
| ***HGF*** | 13.00 ± 0.27 | 9.15 ± 1.20 | 1.00 | 9.00 ± 1.68 | 13.90 ± 0.59 | 1.00 |
| ***CAL 27*** | 12.90 ± 2.56 | 6.35 ± 1.75 | 0.92 | 16.10 ± 0.57 | 33.30 ± 0.65 | 1.04 |
| ***SCC-9*** | 2.85 ± 0.01 | 2.80 ± 0.01 | 4.41 | 16.65 ± 0.71 | 3.65 ± 0.40 | 1.97 |
| ***Detroit 562*** | 3.90 ± 0.13 | 3.50 ± 0.11 | 1.68 | 42.25 ± 0.62 | 18.05 ± 2.63 | 1.30 |
| ***FaDu*** | 5.75 ± 0.06 | 3.85 ± 0.24 | 2.40 | 17.10 ± 2.24 | 21.55 ± 0.73 | 1.09 |
| ***TR146*** | 11.35 ± 0.78 | 4.90 ± 0.42 | 1.05 | 16.75 ± 0.81 | 18.15 ± 1.07 | 0.47 |
| ***Hep-2*** | 11.05 ± 0.32 | 5.15 ± 0.07 | 1.63 | 21.20 ± 0.02 | 37.65 ± 0.36 | 0.32 |
| ***KB*** | 10.50 ± 0.43 | 3.70 ± 0.42 | 1.45 | 25.15 ± 0.33 | 30.30 ± 0.65 | 1.36 |

|  |  | | |
| --- | --- | --- | --- |
|  | ***24h*** | ***48h*** | ***72h*** |
| ***HGF*** | 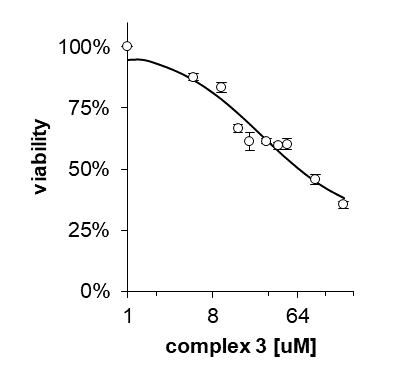 | 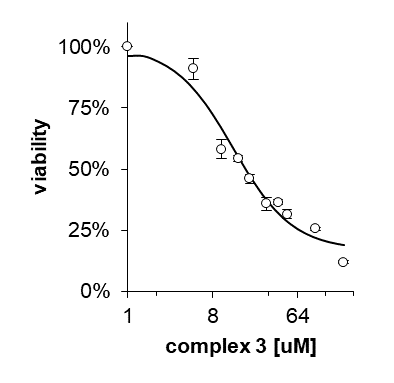 | 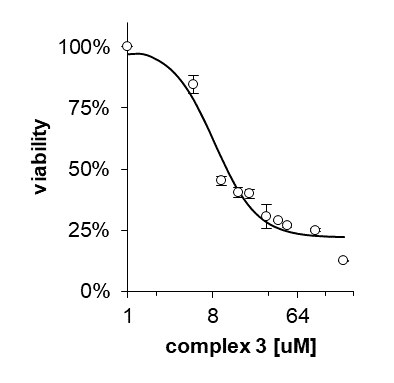 |
| ***CAL 27*** | 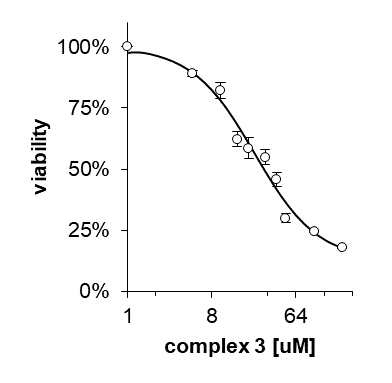 | 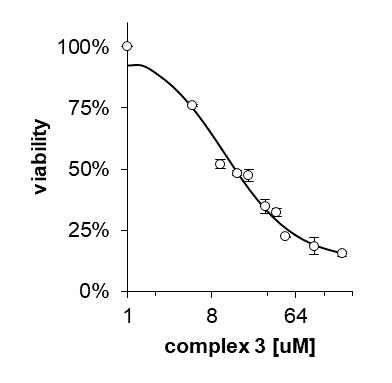 | 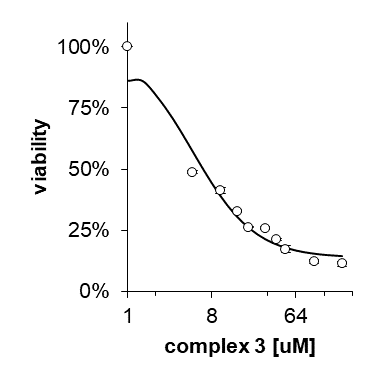 |
| ***SCC-9*** | 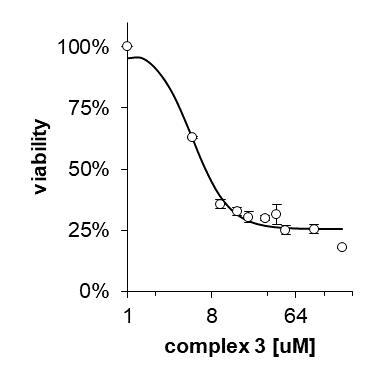 | 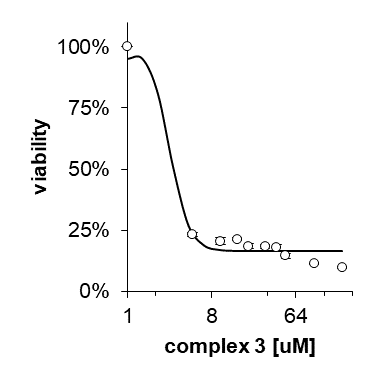 | 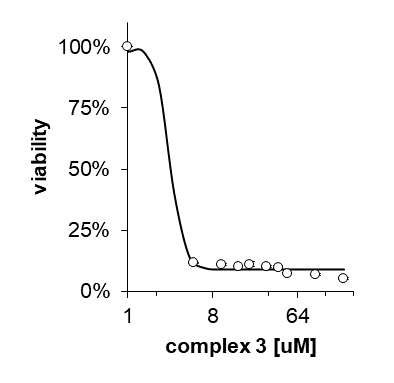 |
| ***Detroit 562*** | 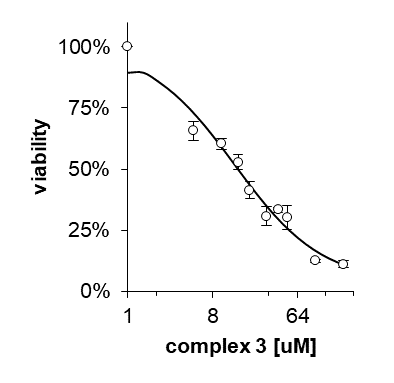 | 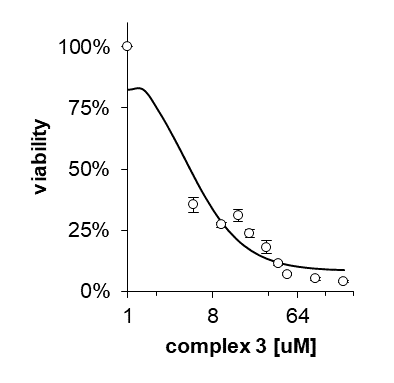 | 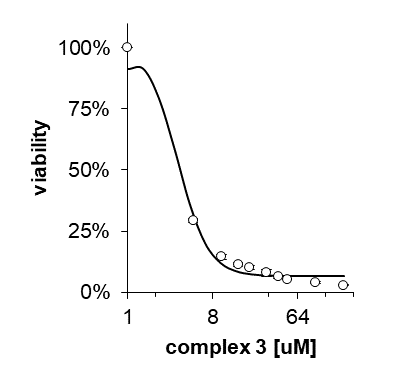 |
| ***FaDu*** | 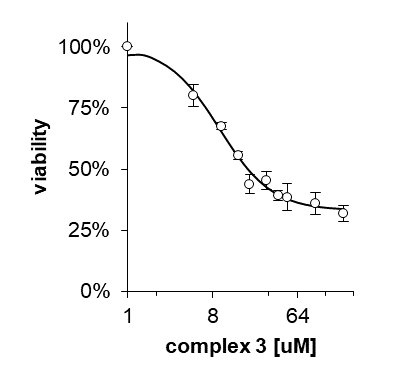 | 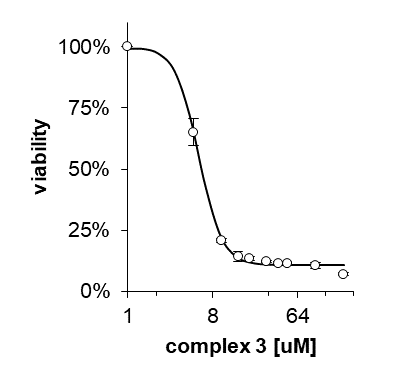 | 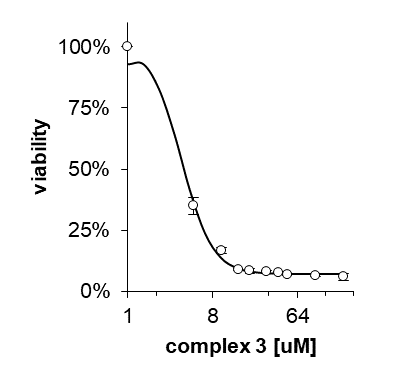 |
| ***TR146*** | 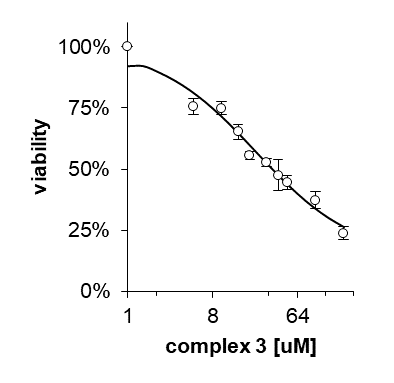 | 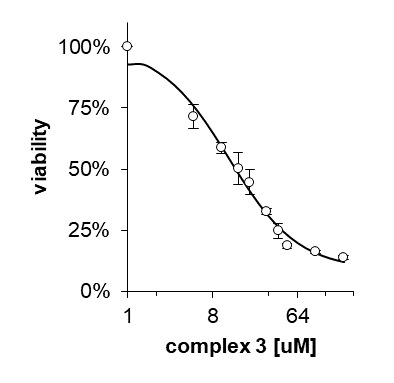 | 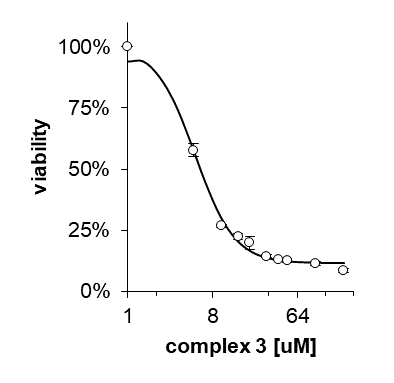 |
| ***Hep-2*** | 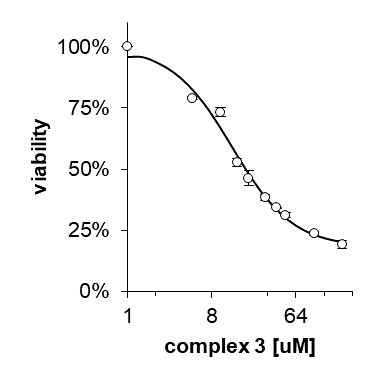 | 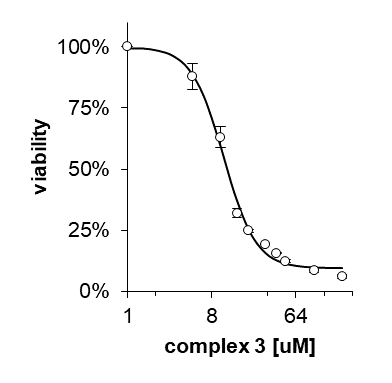 | 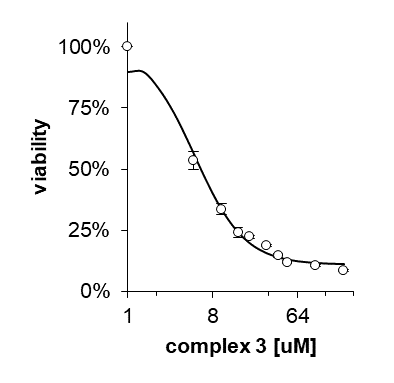 |
| ***KB*** | 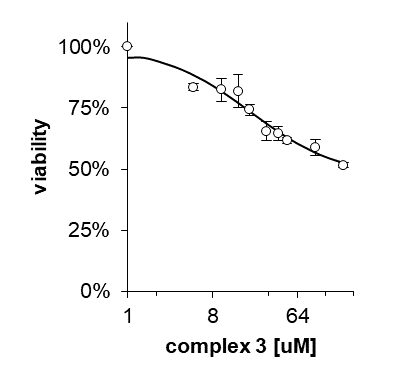 | 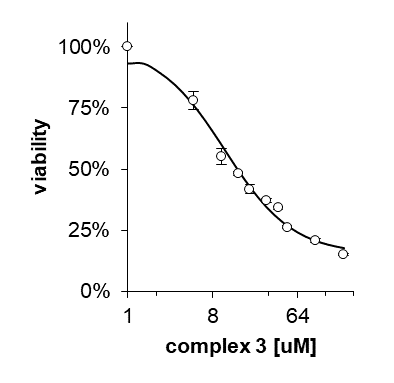 | 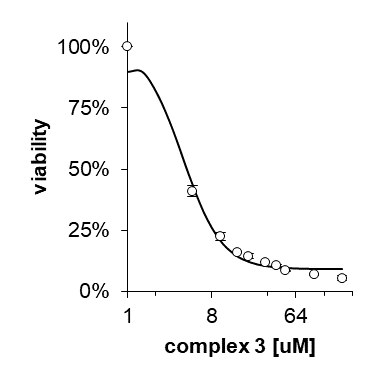 |

|  |  | | |
| --- | --- | --- | --- |
|  | ***24h*** | ***48h*** | ***72h*** |
| ***HGF*** | 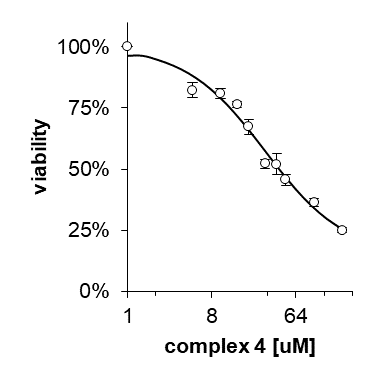 | 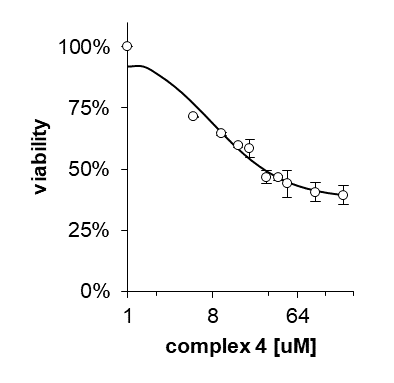 | 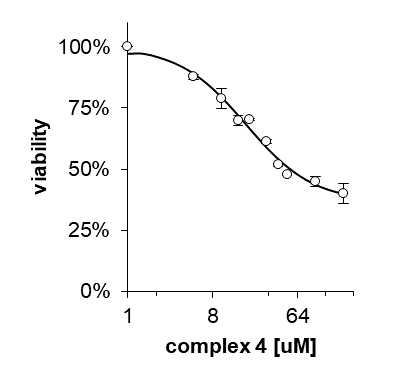 |
| ***CAL 27*** | 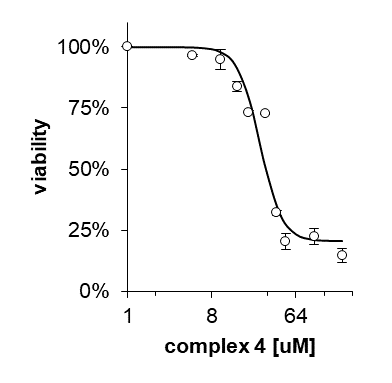 | 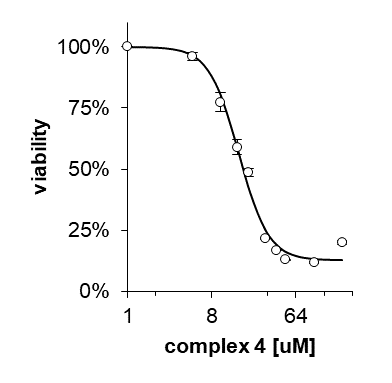 | 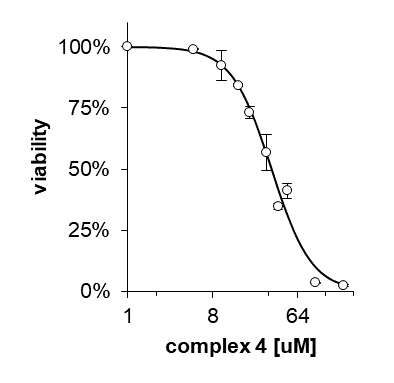 |
| ***SCC-9*** | 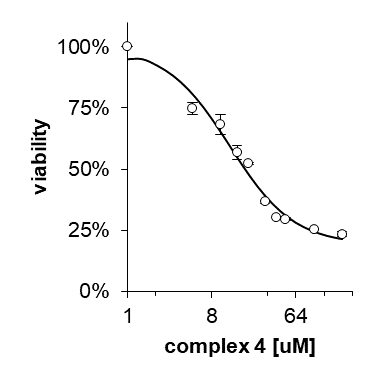 | 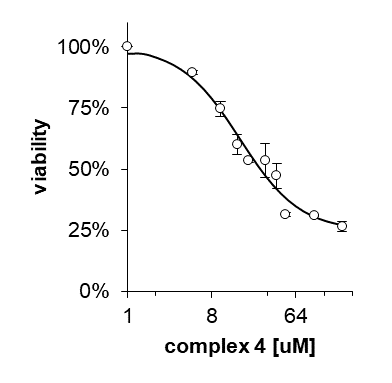 | 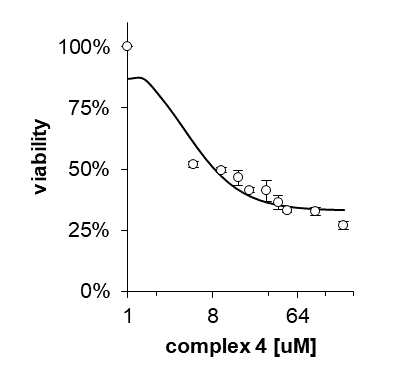 |
| ***Detroit 562*** | 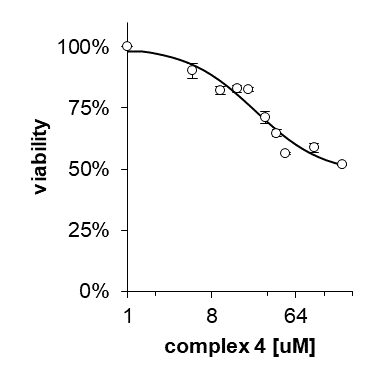 | 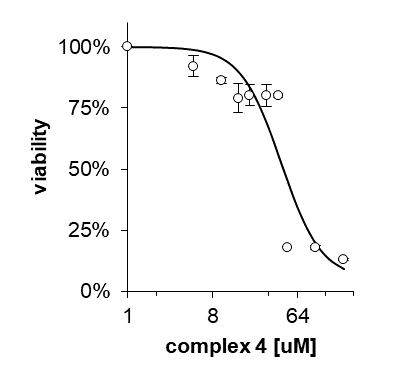 | 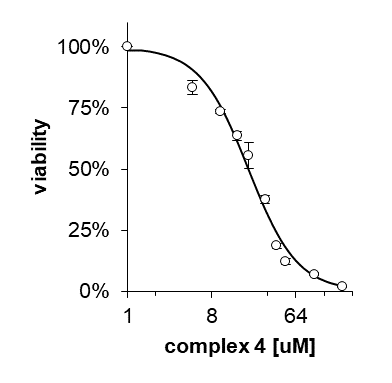 |
| ***FaDu*** | 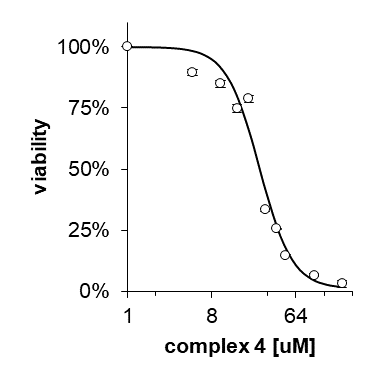 | 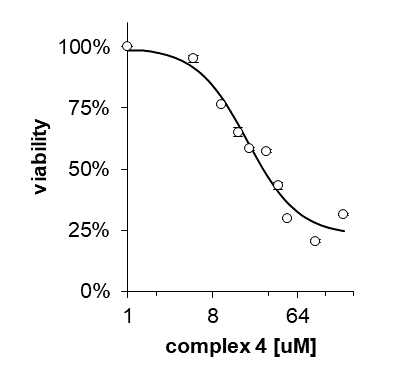 | 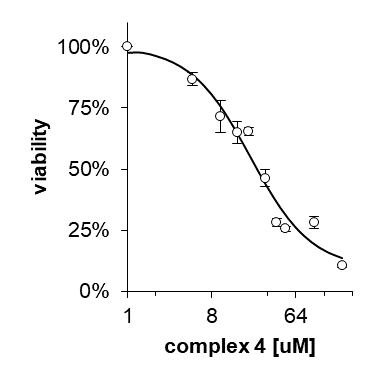 |
| ***TR146*** | 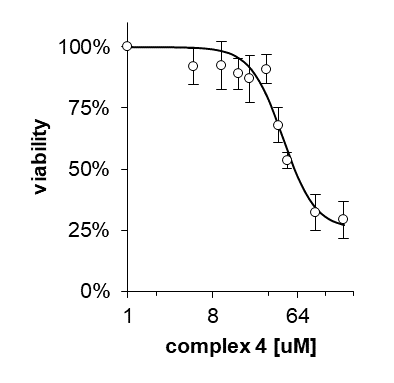 | 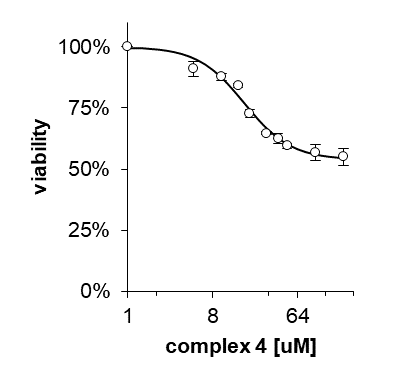 | 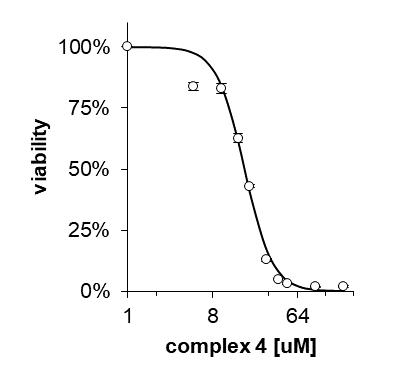 |
| ***Hep-2*** | 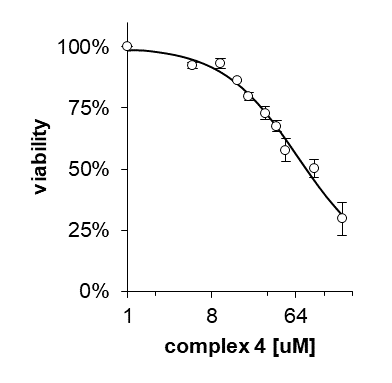 | 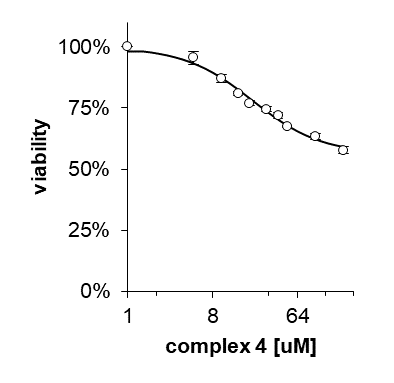 | 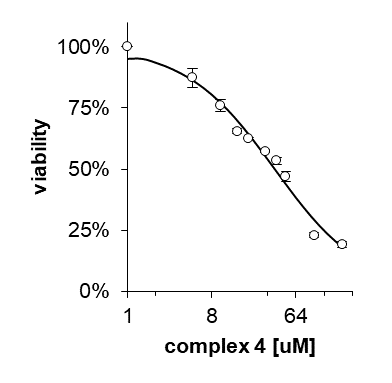 |
| ***KB*** | 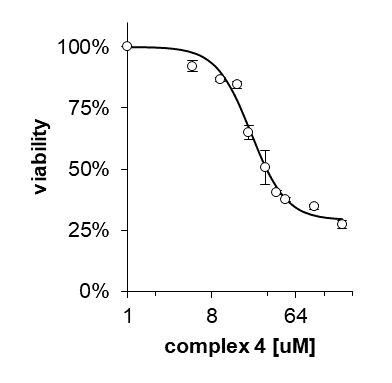 | 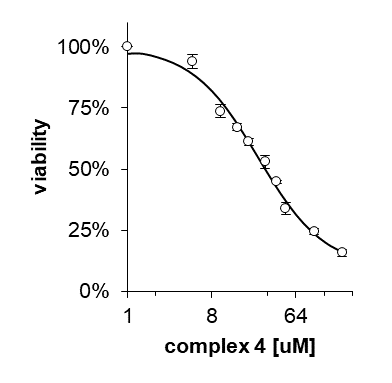 | 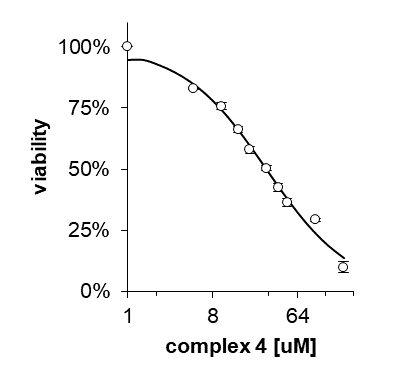 |
|  |  |  |  |

Figure S5 Graphical form of cytotoxicity of complex **3**/complex **4** in normal (HGF) and cancer cells (CAL 27, SCC-9, Detroit 562, FaDu, TR146, Hep-2 and KB).

**Colony formation assay**

|  | **CTR** | **complex 3** | **complex 4** |
| --- | --- | --- | --- |
| ***HGF*** | 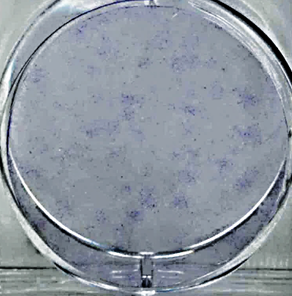 | 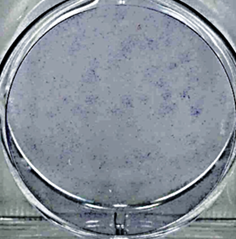 | 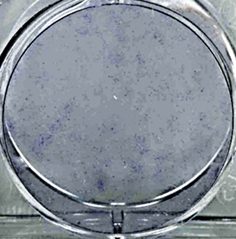 |
| ***CAL 27*** | 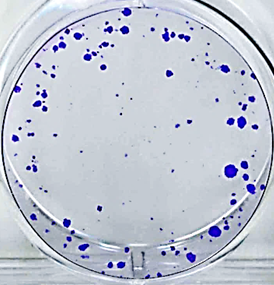 | 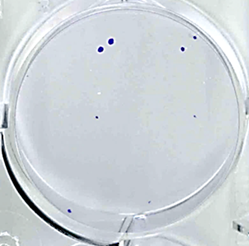 | 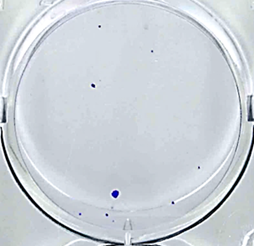 |
| ***SCC-9*** | 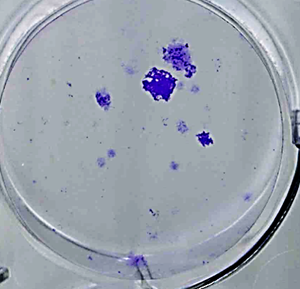 | 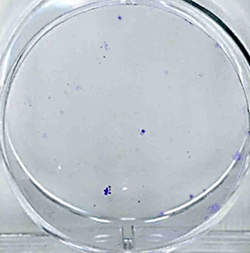 | 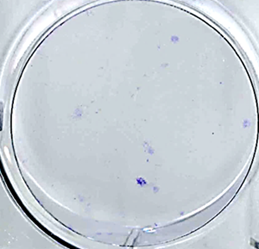 |
| ***Detroit 562*** | 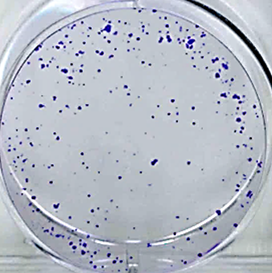 | 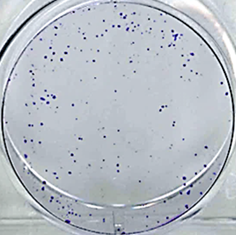 | 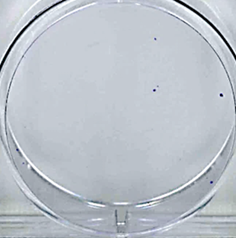 |
| ***FaDu*** | 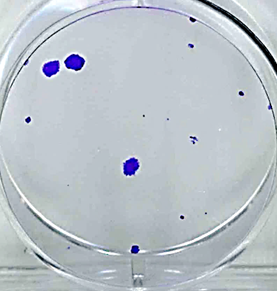 | 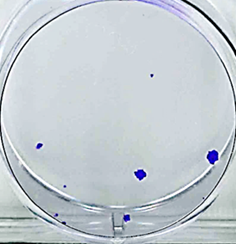 | 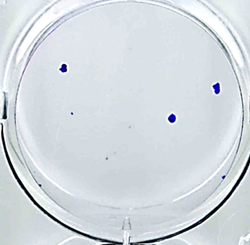 |
| ***TR146*** | 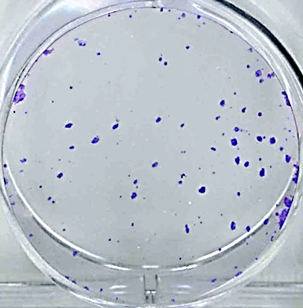 | 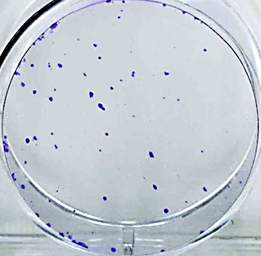 | 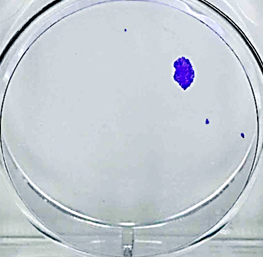 |
| ***Hep-2*** | 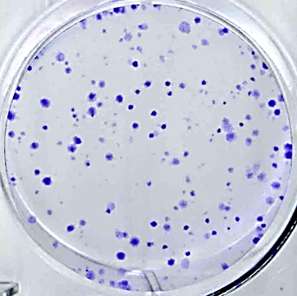 | 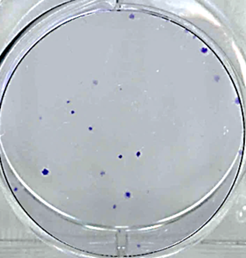 | 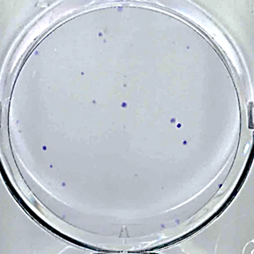 |
| ***KB*** | 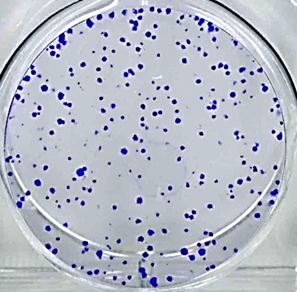 | 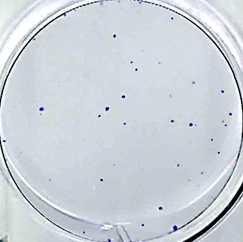 | 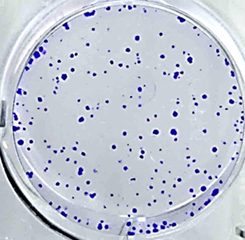 |

Figure S6 CFA was used to determine the ability to form colonies with and without treatment with complex **3**/complex **4**. CFA plates were strained after two weeks incubation in standard conditions.

**Wound healing assay**

|  |  | | **CTR** | **complex 3** | **complex 4** | |
| --- | --- | --- | --- | --- | --- | --- |
|  | | **HGF** | | | |  |
| ***24h*** |  | | 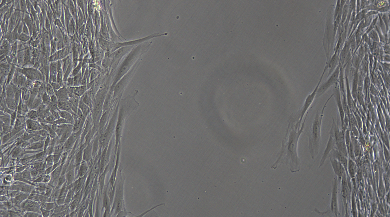 | 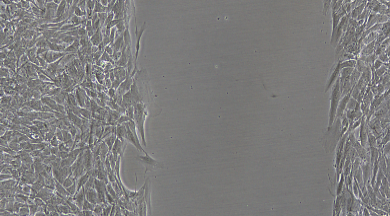 | 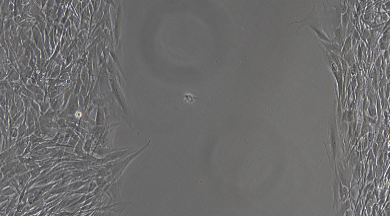 | |
| ***55h*** |  | | 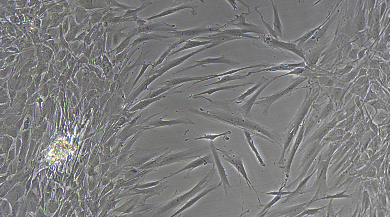 | 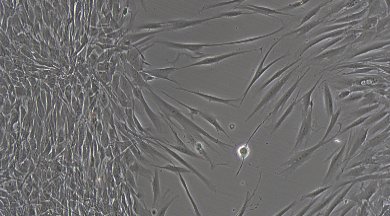 | 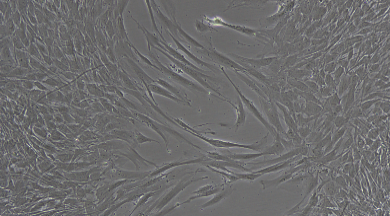 | |
|  | | ***CAL 27*** | | | |  |
| ***24h*** |  | | 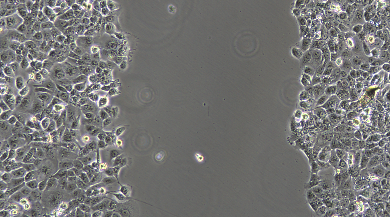 | 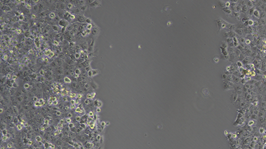 | ***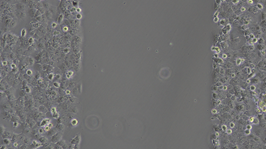*** | |
| ***55h*** |  | | 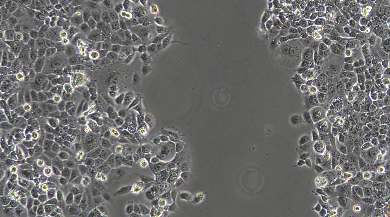 | 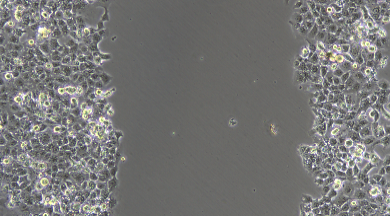 | 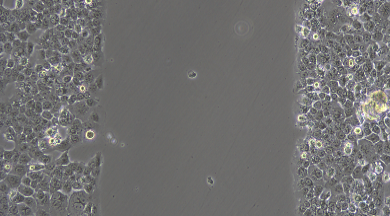 | |
|  | | ***SCC-9*** | | | |  |
| ***24h*** |  | | 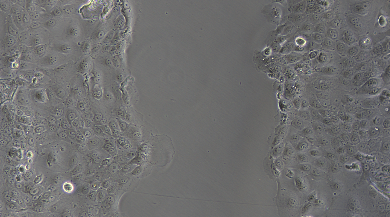 | 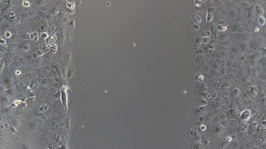 | 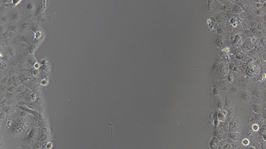 | |
| ***55h*** |  | | 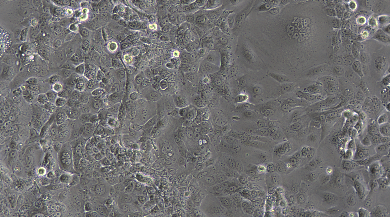 | 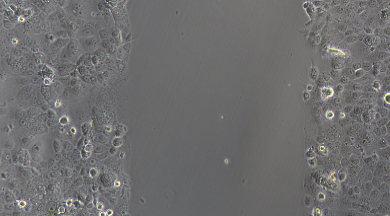 | 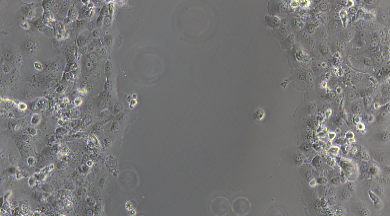 | |
|  | | ***Detroit 562*** | | | |  |
| ***24h*** |  | | 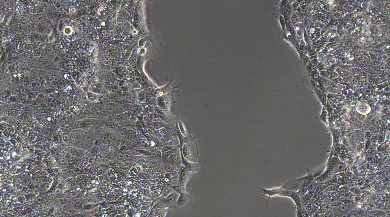 | 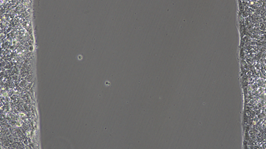 | ***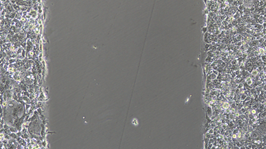*** | |
| ***55h*** |  | | 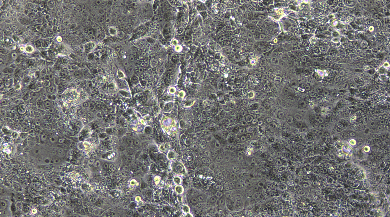 | 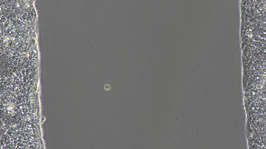 |  | |
|  | | ***TR146*** | | | |  |
| ***24h*** |  | |  |  |  | |
| ***55h*** |  | |  |  |  | |
|  | | ***FaDu*** | | | |  |
| ***24h*** |  | |  |  |  | |
| ***55h*** |  | |  |  |  | |
|  | | ***Hep-2*** | | | |  |
| ***24h*** |  | |  |  |  | |
| ***55h*** |  | |  |  |  | |
|  | | ***KB*** | | | |  |
| ***24h*** |  | |  |  |  | |
| ***55h*** |  | |  |  |  | |

Figure S7 Microscopic imaging of cell migration and area closure after 24h and 55h. Imaging od treated cells (complex **3**/complex **4**) and non-treated cells (control).

| ***HGF*** |  | ***CAL 27*** |  |
| --- | --- | --- | --- |
| ***SCC-9*** |  | ***Detroit 562*** |  |
| ***FaDu*** |  | ***TR146*** |  |
| ***Hep-2*** |  | ***KB*** |  |

*Figure S8 Graphical form of relative wound density of treated cells with complex 3 or complex 4 (the concentration3/complex 4) and non-treated cells (control).*

|  |  |
| --- | --- |
|  |  |
|  |  |
|  |  |

Figure S9 Graphical representation of wound area in time (2-55h) with (complex **3**/complex **4**) without treatment (control) for normal (HGF) and cancer cells (CAL 27, SCC-9, Detroit 562, FaDu, TR146, Hep-2 and KB).

|  | **CTR** | **complex 3** | **complex 4** |
| --- | --- | --- | --- |
| ***HGF*** |  |  |  |
| ***CAL 27*** |  |  |  |
| ***SCC-9*** |  |  |  |
| ***Detroit 562*** |  |  |  |
| ***FaDu*** |  |  |  |
| ***TR146*** |  |  |  |
| ***Hep-2*** |  |  |  |
| ***KB*** |  |  |  |

Figure S10 The wound healing area with and without treatment with complex **3**/complex **4** after 55 h, stained with crystal violet solution.

**ELISA assay IL-6R**

Figure S11 The ELISA IL-6R kit was used to determine the effect of complex **3** and complex **4** on IL-6R compared to native curcumin. Data are presented as the mean ± SEM of four independent experiments (n = 4). Statistical analysis was performed using a one-way ANOVA with Dunnett’s multiple comparison tests. Results are reported as ns = not significant and ****p < 0.0001.

#### **NMR and HRMS spectra**

Figure S12 ^1^H NMR (400 MHz, DMSO-d_6_) spectrum of compound **1**.

Figure S13 ^13^C{^1^H} NMR (101 MHz, DMSO‑d_6_) spectrum of compound **1.**

Figure S14 ^1^H NMR (400 MHz, DMSO-d_6_) spectrum of compound **2**.

Figure S15 ^13^C{^1^H} NMR (101 MHz, DMSO‑d_6_) spectrum of compound **2.**

Figure S16 ^1^H NMR (400 MHz, DMSO-d_6_) spectrum of complex **3**.

Figure S17 ^13^C{^1^H} NMR (101 MHz, DMSO‑d_6_) spectrum of complex **3.**

Figure S18 ^1^H NMR (400 MHz, DMSO-d_6_) spectrum of complex **4**.

Figure S19 ^13^C{^1^H} NMR (101 MHz, DMSO‑d_6_) spectrum of complex **4.**

Figure S20 Full NMR assignment and diagnostic 2D correlations observed for complex **3** and complex **4**. The green numbers indicate the atom numbering for the complexes.

Figure S21 HRMS spectrum of complex 3; for C45H46Cl4N2O2Ru2, the ion observed corresponds to the fragment [M−(Ru(p-cymene)Cl2)] at m/z = 685.0956.

Figure S22 HRMS spectrum of complex **4**; for Chemical Formula: C_37_H_42_Cl_4_N_2_ORu_2_ the ion observed corresponds to the fragment [M − (Ru(p-cymene)Cl_3_)] at m/z = 533.0929.

#### **UV-VIS characterization**

*Figure S23 UV–Vis spectra of complex 3 and complex 4 (left), including the corresponding calibration curves (right).*
